# Modulation of microglia activation by the ascorbic acid transporter SVCT2

**DOI:** 10.1016/j.bbi.2024.07.003

**Published:** 2024-07-06

**Authors:** Amanda L. Marino, Tonia S. Rex, Fiona E. Harrison

**Affiliations:** a Vanderbilt Brain Institute, Vanderbilt University, Nashville, TN, United States; b Division of Ophthalmology & Visual Sciences, Vanderbilt University Medical Center, Nashville, TN, United States; c Division of Diabetes, Endocrinology, and Metabolism, Department of Medicine, Vanderbilt University Medical Center, Nashville, TN, United States

**Keywords:** Neuroinflammation, Microglia, Ascorbate, Vitamin C, Mouse, HMC3, Cytokines

## Abstract

Neuroinflammation is a major characteristic of pathology in several neurodegenerative diseases. Microglia, the brain’s resident myeloid cells, shift between activation states under neuroinflammatory conditions, both responding to, but also driving damage in the brain. Vitamin C (ascorbate) is an essential antioxidant for central nervous system function that may have a specific role in the neuroinflammatory response. Uptake of ascorbate throughout the central nervous system is facilitated by the sodium-dependent vitamin C transporter 2 (SVCT2). SVCT2 transports the reduced form of ascorbate into neurons and microglia, however the contribution of altered SVCT2 expression to the neuroinflammatory response in microglia is not well understood. In this study we demonstrate that SVCT2 expression modifies microglial response, as shown through changes in cell morphology and mRNA expression, following a mild traumatic brain injury (mTBI) in mice with decreased or increased expression of SVCT2. Results were supported by *in vitro* studies in an immortalized microglial cell line and in primary microglial cultures derived from SVCT2-heterozygous and transgenic animals. Overall, this work demonstrates the importance of SVCT2 and ascorbate in modulating the microglial response to mTBI and suggests a potential role for both in response to neuroinflammatory challenges.

## 1. Introduction

Neuroinflammation in the central nervous system is a key feature of many neurological disorders including traumatic brain injury (TBI) as well as neurodegenerative diseases such as Alzheimer’s disease and related dementias (Smith et al., 2013; Chiu et al., 2016; Simon et al., 2017; Dinet et al., 2019; Wofford et al., 2019). It is estimated that about 1.7 million people sustain a TBI each year, and experience TBI-associated cognitive and behavioral changes following injury (Langlois et al., 2006). The majority of injuries are mild (mTBI) but even mild injuries can give rise to cognitive, behavioral, and motor impairments as well as an increased risk of developing neurodegenerative disorders (Caye et al., 2018; Livingston et al., 2020; Mielke et al., 2022). Considerable evidence has shown that neuroinflammation is at least partially regulated by microglial cells, the resident immune cells of the brain (Subhramanyam et al., 2019; Muzio et al., 2021; Shao et al., 2022). Microglia change their shape and function in response to inflammatory signals, such as reactive oxygen species (ROS) in the microenvironment. While these signals are necessary for microglia to respond to different pathological states by preventing further damage, chronic microglial activation can lead to the progression of neuronal dysfunction (Aguzzi et al., 2013; Li and Barres, 2018). Under oxidative stress, cytokines and other proinflammatory signals are produced by microglia and released into their environment, which in turn promotes a reactive phenotype in other microglia (Smith et al., 2012). This unchecked inflammatory state has been the target of drug intervention approaches due to the ubiquity of neuroinflammation across disease states, however, there are still many unknowns regarding mediators of microglial dysregulation that can help to prevent such damage (Mullard, 2018; Maurya et al., 2021).

As the most abundant antioxidant in the brain, vitamin C (ascorbate, ASC) is recognized for its ability to scavenge ROS and prevent oxidative stress in the brain microenvironment (Harrison and May 2009). In most animals, ASC is synthesized in the liver, however humans and other non-human primates have lost this ability over the course of evolution through the loss of the *gulonolactone oxidase* gene responsible for the final step in ASC synthesis. Therefore, humans and other primates rely on dietary intake of ASC and are at risk of depletion and deficiency. ASC is found in its oxidized form as dehydroascorbic acid (DHA). DHA enters cells by facilitated transport through glucose transporters (GLUT1, GLUT3), whereas ASC is transported throughout the body by the two sodium-dependent vitamin C transporters, SVCT1 and SVCT2 (Rumsey et al., 1997; May 2012). SVCT1 mainly transports ASC in the kidney, liver, and intestines, whereas SVCT2 transports ASC in highly metabolically active tissues including the brain, eye, and lung (Tsukaguchi et al., 1999; Savini et al., 2008; Harrison and May 2009). Less well characterized is a more specific role for ASC uptake within microglia, as the majority of research to date has focused on ASC uptake in neurons. SVCT2 was identified in microglia by Mun and colleagues, suggesting a role for ASC or SVCT2 in microglial function (Mun et al., 2006). By preventing excess generation of ROS, ASC can mediate neuroinflammation through its antioxidant properties and thereby diminish the release of cytokines, chemokines, and other signals (Harrison and May 2009; Portugal, 2024). More recently it has been shown that ASC mediates morphology changes, and expression of inflammatory mediators through the NF-κB pathway *in vitro* (Portugal et al., 2017; Portugal et al., 2017).

In the present study, we sought to determine the effects of under- or over-expression of SVCT2 on microglial activation and neuroinflammation after mTBI. We used a closed-head blast mTBI to induce neuroinflammation in *in vivo* mouse models of altered ASC transport. Although some studies have examined sensorimotor gating following mTBI, these studies have characterized changes from 24 h up to weeks later (Wiley et al., 1996; Arciniegas et al., 2005; Washington et al., 2012; Heldt et al., 2014; Sinha et al., 2017; Browne et al., 2022). Significant changes in inflammatory proteins and genes have been observed from 8 to 24 h in a rodent weight drop model of mild TBI, although many of these changes extend to 96 h post injury with some evident as far as 1.5 months post injury (Tweedie et al., 2020; Bolte and Lukens, 2021). In female mice exposed to an LPS-model of cystitis gene changes were observed from 30 mins to 24 h with the gene cluster representing NF-κB pathways peaking at 4 h (Cluster 12, Fig. 6; Saban et al., 2001). Although some studies have used shorter time points (e.g. 3.5 h) with TBI as the inflammatory challenge, behavioral changes and sex differences were not always explicitly assessed (Feng et al., 2010). We wanted to examine an earlier time point which would help suggest a timeline for potential therapeutic interventions following mTBI. The endotoxin lipopolysaccharide (LPS) is a surface membrane component of gram-negative bacteria and was used as an inflammatory stimulus in the *in vitro* models. Both mTBI *in vivo* and LPS *in vitro* are known to induce neuroinflammation and the activation of the NF-κB pathway (Batista et al., 2019; Verboon et al., 2021). We hypothesized that overexpression of SVCT2 would have a protective effect, while partial knockdown would increase vulnerability to injury as indicated by microglial morphology changes as well as downstream proinflammatory cytokines. We also sought to clarify the extent to which observed effects were specific to ASC and its transporter, or whether it could be attributed to more general antioxidant properties.

## 2. Materials and methods

### 2.1. Experimental animals

Brain ASC is tightly regulated by SVCT2 to maintain physiological levels in brain despite changes in dietary intake or hepatic synthesis except in cases of prolonged depletion. Two separate genetically engineered mouse lines that differed in their expression of SVCT2 were used for these studies. All mice carried functional copies of the *L-gulono-lactone oxidase* gene and could thus synthesize ASC in the liver and so were not provided with additional ASC supplementation. Mice were housed under a 12 h light/dark cycle with food and water available ad libitum and were 12–16 weeks old when used for TBI experiments. The use of these mouse models was approved by the Vanderbilt University Institutional Committee for Animal Use and Care and was conducted in accordance with NIH guidelines.

#### Line one:

SVCT2^+/+^ (wild-type) and SVCT2^+/−^ (heterozygous knockout). SVCT2^+/−^ mice are heterozygous for the SVCT2 gene, Slc23a2, and have 50 % of the transporter expression of a normal wildtype mouse leading to ~30 % decrease in brain ASC levels (Harrison et al., 2010). SVCT2^+/+^ littermates were used as controls. Full knockouts (SVCT2^−/−^) do not survive past birth. These mice were bred and maintained in-house with wild-type dams (SVCT2^+/+^) mated with SVCT2^+/−^ males to minimize the potential impact of ASC depletion in-utero due to altered SVCT2 expression in placenta.

#### Line two:

SVCT2-Tg^BAC−^ (wild-type), SVCT2-Tg^BAC+^ (transgenic overexpression). SVCT2-Tg^BAC+^ inherit a bacterial artificial chromosome (BAC) transgene for SVCT2 and therefore have a higher expression of the transgene than native copies leading to up to 2-fold increase in SVCT2 expression and ASC in SVCT2-dependent organs including brain (Harrison et al. 2012). The SVCT2-Tg^BAC−^ littermates have WT-levels of SVCT2 expression and are used as controls for the SVCT2-Tg^BAC+^ mice. These mice were bred and maintained in-house with wild-type dams mated with transgenic males (SVCT2-Tg^BAC+^).

**Fig. 1.**
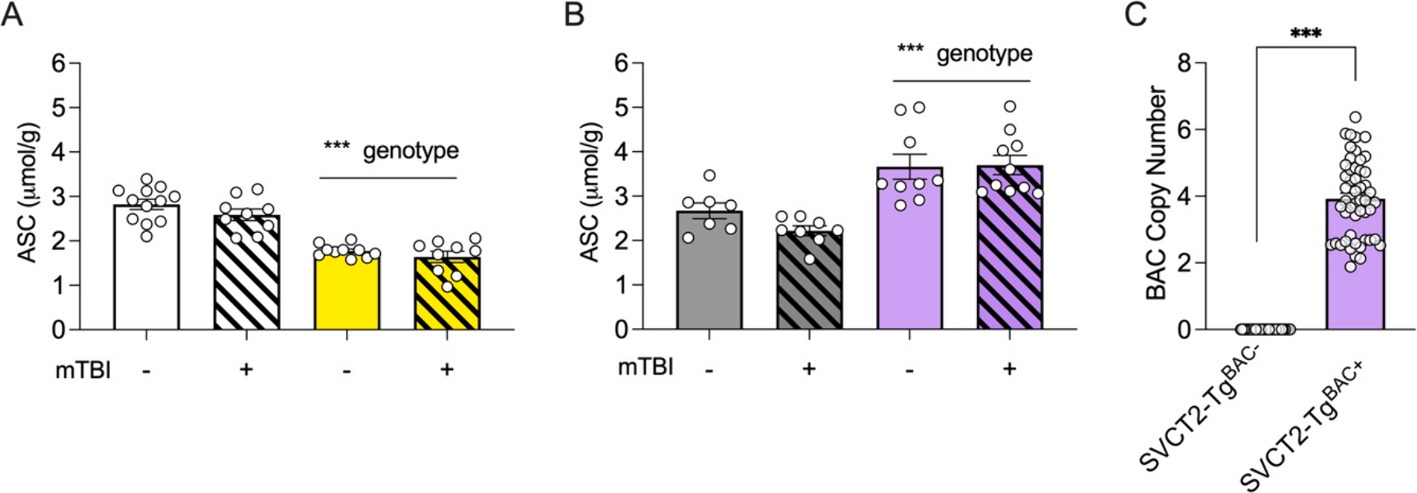
Characterization of brain ASC in SVCT2 mouse models. **(A-B)** ASC in the cortex was decreased in SVCT2^+/−^ mice compared to wild-type littermates **(A)** and increased in of SVCT2-Tg^BAC+^ compared to SVCT2-Tg^BAC−^ controls **(B)**. **(C)** Copy number of SVCT2 in SVCT2-Tg^BAC+^ mice was higher than SVCT2-Tg^BAC−^ mice. Data shown are for a representative sample of mice used in the studies described here. Data are presented as ± S.E.M with main effects noted above graphs. n = 4–6 male and female mice in each group for A and B. ***=p < 0.001.

All experiments were conducted in accordance with the NIH Guide for the Care and Use of Laboratory Animals. Genotyping was performed for all mice from a tail snip at 21 days by Transnetyx. SVCT2-Tg mice were checked for expression level of the BAC gene, which indicates additional copies of SVCT2. The relative expression amounts varied between mice as determined by semi-quantitative PCR performed by Transnetyx (Fig. 1C). The BAC gene sequences provided to Transnetyx for the SVCT2-Tg BAC gene are: Forward: 5′ GGAAATCGTCGTGGTATTCACTC 3′. Reverse: 5′ TCCCAATGGCATCGTAAAGAAC 3′ (Harrison et al., 2012).

### 2.2. Mouse mild blast injury treatment (mTBI)

All mice were 12–16 weeks of age at the time of injury. Mice were randomly assigned to receive a mTBI or sham injury. We performed a focal mTBI modified from the methods previously described, or a sham injury (Heldt et al., 2014). Prior to blast exposure, mice were anesthetized using 3 % isoflurane in oxygen for ~5 min and were maintained at 2.5 % isofluorane in oxygen throughout the blast or sham exposure. Mice were then placed in a specially designed mouse restraint device secured within a foam rubber sleeve with the frontal cortex positioned facing a 20 mm opening in the tube. The inner tube was slid into a 44 mm outer diameter plexiglass pipe with a 7.5 mm hole bored into the midpoint. The frontal cortex target region was aligned to a modified paintball gun barrel opening at a 10 mm distance from barrel tip. This allows the blast to target the frontal cortex only while shielding the rest of the mouse from the blast. Rather than a single blast exposure as utilized previously (Heldt et al., 2014) mice were exposed to three repeated blasts of 37–40 PSI to the frontal cortex with an inter-blast interval of 1 s. The blast pressure output was calibrated before and after each mouse exposure. Mice that received a sham injury were anesthetized and placed into the plexiglass tube apparatus but neither head nor body was aligned to the gun barrel output, so they were exposed only to the noise of the blasts.

### 2.3. Pre-pulse inhibition of the acoustic startle response (PPI)

PPI was measured 4 h following the mTBI. Mice were secured in a small, clear acrylic cylinder mounted on a white acrylic base. The acrylic base was secured to a startle platform detector housed within an acoustic chamber insulated with sound attenuating foam (ENV-022S, MED Associates). Startle and pre-pulse stimuli were delivered using Startle-PPI Pro Series software. Testing consisted of 6 different trial types (null, 0 dB; startle only at 120 dB; pre-pulse trials of 70, 76, 82, 88 dB each followed by a startle 120 dB) repeated nine times in a pseudo-randomized order (n = 7–10 males or females per group). The startle inhibition (PPI) is calculated from the group’s own baseline startle so can be compared even when baseline startle values are different. Animals with a negative or zero value for startle response were excluded from analyses, therefore only animals with presumed normal hearing and the ability to startle were included.

### 2.4. Ascorbate quantification

Ascorbate was measured in liver and cortical tissue from the area of injury. Samples were prepared by homogenizing in 10 μl extraction buffer (3.5:1 25 % w/v metaphosphoric acid: phosphate-EDTA, pH 8.0) per mg of wet tissue to normalize by weight. Samples were centrifuged at 10,000 rpm, and the clear supernatant was transferred to a fresh tube. Concentrations of ASC were measured at 1:100 dilution for cortical tissue and 1:50 dilution for liver tissue with ion pair HPLC as previously described (May et al., 1998; Harrison et al., 2008; Consoli et al., 2021).

### 2.5. RNA Isolation, cDNA synthesis, and RT-qPCR

Total RNA was extracted from fresh frozen cortical tissues and immortalized and primary cells.

#### Brain tissue.

At the time of euthanasia, brains were rapidly removed and dissected and immediately frozen on dry ice. Total RNA was extracted from the cortical samples using the RNeasy Plus Mini kit (Qiagen, cat #74134) according to manufacturer’s specifications. RNA integrity was determined using NanoDrop 2000 (Thermo Scientific, cat # ND-2000). All RNA samples included had a A260/280 ratio > 1.8.

#### Cells.

Cells for qPCR were grown in 6-well plates and were treated 24 h prior to collection for RNA extraction. Media was removed, and 350 μL of Buffer RLT Plus with 10 μL β-mercaptoethanol (β-ME) added per 1 mL of Buffer RLT plus. Well contents were pipetted into Eppendorf tubes. RNA was extracted as above. cDNA for both cells and tissue was made using the High-Capacity cDNA Reverse Transcription Kit according to manufacturer’s instructions (Applied Biosystems, cat #4368814). qPCR was performed using the BioRad CFx96 thermocyclers in the Vanderbilt Molecular Cell Biology Resource Core based using PowerUp SYBR Green Master Mix (Thermofisher, #A25742) to assess nuclear factor kappa-light-chain enhancer of activated B cells (NF-κB) and proinflammatory cytokines interleukin 6 (IL-6) and interleukin 1 beta (IL-1β). Relative expression was estimated using the delta-delta Ct method and were normalized to the geomean of β-Actin and GAPDH serving as the reference genes. All samples were run in triplicate.

### 2.6. Western blot

Protein lysates were prepared by homogenizing frozen cortical tissue in 250 μL of Pierce RIPA lysis buffer (Thermo Fisher Scientific, #89900) and protease inhibitor cocktail tablets (cOmplete Tablets, Mini EDTA-free, Roche, #04693159001). Protein concentration was measured using a standard BCA assay protocol (Pierce BCA Protein Assay Kit, Thermo Fisher Scientific, #23225). Samples were denatured with NuPage LDS sample and reducing agent (Thermo Fisher Scientific #NP0007, #NP0009). Samples were loaded into Bolt^™^ 4–12 % Bis-Tris Plus gels (Thermo Fisher Scientific, #NW04120BOX) with 20 μg protein per well. Samples were transferred to PVDF membranes using the iBlot2^™^ system (Thermo Fisher Scientific, cat # IB24001). Following transfer, membranes were blocked for 30 min with 5 % nonfat milk in tris-buffered saline with 0.1 % Tween-20 (TBST). Blots were incubated overnight with one of the following primary antibodies in 5 % milk in TBST [Mouse anti- Il-1β [1:1000], Cell Signaling Technologies #12242; mouse anti-actin [1:1000] Santa Cruz Biotechnology #sc-47778] at 4 °C on a rotator. The next day following primary antibody incubation, blots were washed 3x for 5 min in TBST then incubated for 2 h with horseradish peroxidase (HRP) conjugated secondary antibody [HRP-anti-mouse, [1:5000] Promega #W402B]. Protein bands were visualized using chemiluminescence (Western Lighting Plus ECL, Perkin Elmer #21059). Membranes were stripped with Restore Stripping Buffer (Thermo Fisher Scientific, #21059) before probing subsequent proteins. Protein bands were quantified using ImageJ (imagej.nih.gov). Each protein band was normalized to its own actin control, then to average wild-type (SVCT2^+/+^ or SVCT2-Tg^BAC−^) sham control for that blot.

### 2.7. Microscopy and Morphometric analysis of microglia

#### Brain slices.

24 h following blast or sham injury, mice were anesthetized with an overdose of isofluorane and transcardially perfused with saline. Brains were removed and the anterior half of the brain was immersion-fixed in 4 % paraformaldehyde for 24 h, moved to 30 % sucrose solution for 48 h, then stored in PBS at 4 °C until sliced. The posterior half of the brain was dissected to obtain cortex, hippocampus, and cerebellar tissue and frozen at −80 °C. Anterior brains were sliced coronally at 35 μm thickness on a sliding freezing microtome. For epitope retrieval, free-floating sections were covered in sodium citrate buffer and heated to 95 °C for 45 min. Slices were washed with 1XPBST three times and blocked in 5 % BSA/1XPBST for 2 h at room temperature. Sections were incubated with primary antibody diluted in blocking reagent overnight at 4 °C (rabbit anti-IBA1 [1:1000], Wako Diagnostics #019–19741). The next day, slices were washed in 1X PBST three times and incubated with goat anti-rabbit Alexa Fluor 488 superclonal recombinant secondary antibody (goat anti-rabbit, 1:250, Invitrogen #A27034) for 2 h at room temperature. Slices were mounted with ProLong Diamond Antifade Mountant with DAPI (Thermofisher Scientific #P36962). Using a confocal microscope in the Vanderbilt CISR core (Zeiss, LSM 710) z-stacks were taken in the frontal cortex at the site of injury. All z-stack images were imported to FIJI and the sliding parabaloid background subtraction was used to remove autofluorescence, and contrast was enhanced to 0.35 % saturated pixels, images were saved as tiff files. The tiff files were imported into IMARIS 10.0 3D analysis software (Oxford Instruments, Concord, MA) where the semi-automated analysis was performed. Morphometric analysis was performed on the IBA1 positive microglia whose processes were entirely within the 3D Z-stack (n = 6 microglia/mouse per group, 3–5 male or female mice per group). Cell sphericity was quantified using the surfaces feature and processes were quantified using the automated filament tracing to determine process length and number of branch points (Morrison and Filosa, 2013).

#### Primary Cells.

Primary microglial cells for imaging were grown in Clipmax 10 cm^2^ clip flasks (TPP, cat #70010). 24 h following experimental treatments media was removed and cells were covered with 4 % PFA and placed on a room temperature rocker for 30 min. After 30 min flasks were washed 3X with 1XPBS, and the cells were covered with 5 % BSA in PBS to block for 1 h. Cells were covered in IBA-1 primary antibody (rabbit anti-IBA1 [1:1000], Wako Diagnostics #019–19741) overnight at 4 °C. The next day, the clipflasks were washed with PBS and covered in goat anti-rabbit Alexa Fluor 488 superclonal recombinant secondary antibody (goat anti-rabbit, 1:250, Invitrogen #A27034) for 60 min on a room temperature rocker, protected from light. They were rinsed in PBS and coverslips were mounted using ProLong Diamond Antifade Mountant with DAPI (Thermofisher Scientific #P36962). Cells were imaged using a confocal microscope (Zeiss LSM710) in the Vanderbilt CISR core. Z-stack images were analyzed for sphericity, number of branching points and process length using IMARIS 10.0 3D analysis software (Oxford Instruments, Concord, MA).

### 2.8. Primary microglial isolation

For primary microglial cultures, cortex was removed from P0-P2 pups from SVCT2^+/+^ × SVCT2^+/−^ and SVCT2-Tg^BAC−^ × SVCT2-Tg^BAC+^ mice, dissected and grown in mixed-glial cultures according to previously published protocols (Lian et al., 2016). Briefly, neonatal pups were decapitated, the skull was cut posterior to anterior and brain was isolated and put into dissection media. The meninges were removed, and brains were minced into small pieces using scissors. Brain pieces were transferred to a 50 mL conical tube with 2.5 % Trypsin and incubated for 15 min in a 37 °C water bath. Trypsin inhibitor and DNase were added to the tube and centrifuged for 5 min at 400g. The supernatant was aspirated, and the pellet was resuspended in warmed culture media. Brains from each pup were plated individually into separate T25 flasks until confluent, then split to 6 well plates. Tails from the pups were retained for genotyping. The flasks were maintained in DMEM with 10 % FBS and 1 % Pen/Strep in a CO_2_ cell culture incubator with 5 % CO_2_, at 37 °C, 100 % humidity. Media was changed the following day, then microglia were separated out 5 days later by manual force detachment. Primary microglia were treated with media control (DMEM with 10 %FBS and 1 % Pen/Strep), ASC (10 μM Na-L-ASC and 100 μM 2-Phosphoascorbic Acid (2PA)), 100 ng/mL of lipopolysaccharide (LPS, Millipore Sigma, #L2880), or 100 ng/mL LPS and ASC made up in media for 24 h.

### 2.9. HMC3 cells treatment

The immortalized human fetal brain-derived microglia cells (HMC3) cells were a kind gift from Dr. Aqeela Afzal (Vanderbilt University Medical Center). HMC3 cells were chosen as a standard and reproducible model for confirmation of SVCT2–related changes that would reflect what occurs in humans. Cells were maintained in DMEM with 10 % FBS and 1 % Pen/Strep in an incubator maintained at 37 °C, 5 % CO_2_ and 100 % humidity. Cells were treated with 1 μg/mL of LPS (Millipore Sigma, #L2880, Lipopolysaccharides from Escherichia coli O55:B5) with and without one of the following antioxidants: ASC (10 μM Sodium-L-Ascorbate (Sigma Aldrich, #A4034) and 100 μM 2-Phospho-L-Ascorbate trisodium salt (Sigma Aldrich, #49752), 10 mM glutathione (GSH, reduced Thermofisher Scientific, #78259) or JS0208MD (0.03 μg/μL) for 24 h. Cells did not have ASC in the absence of experimental supplementation. GSH was given as a comparison antioxidant that is also closely linked with ASC recycling. JS0208MD is a nutritional botanical extract powder containing very high ASC content in addition to other natural compounds. ASC content in the JS0208MD compound was matched to the ASC given alone (100 μM) to establish whether observed effects of multiple fruit-derived antioxidants would be additive.

### 2.10. HMC3 cell viability

Cell viability was determined using the WST-1 Cell Proliferation Assay (Abcam, USA, #ab65473) and run according to manufacturer’s specifications. Briefly, cells were plated into a 96-well plate for 24 h before treatment with media with LPS and/or respective antioxidant. 24 h after treatment, 10 μL of the WST-1/ECL reagent was added to each well and cells were kept at 37 °C and 5 % CO_2_ for 4 h_._ Plates were shaken on an orbital shaker for 1 min. Absorbance was measured on a microplate reader at 450 nm with a reference wavelength of 650 nm.

### 2.11. Data and Statistics

Data are presented in figures as mean ± S.E.M. unless otherwise stated. All data were analyzed using GraphPad Prism version 10 (GraphPad Software, San Diego, CA, USA) or SPSS version 29 (IBM Statistics). For all analyses we only compared SVCT2^+/−^ to SVCT2^+/+^ littermate controls, or SVCT2-Tg^BAC+^ to SVCT2-Tg^BAC−^ littermate controls since each mouse line is bred independently and the impact of different parental SVCT2 genotype during development on inflammatory response is not known. Further, although each line is periodically refreshed by reintroducing mice from the original background strain, strain drift over several generations of breeding may also alter inflammatory response. Group sizes were based on data collected during similar earlier studies using mice that underwent the same blast procedure and testing at 4 h post injury and calculated to provide power > 0.8 with p < 0.05. We anticipated sex differences in PPI data which we have observed in previous studies in other genotypes, therefore, we analyzed the two sexes separately in a repeated measures 3-way ANOVA (2 genotype × 2 mTBI condition × 4 prepulse decibel strength) followed by Tukey’s corrections for multiple comparisons. Animals with a negative or zero value for startle response were not included in analyses to ensure that only animals with normal hearing and the ability to startle were included. Since there were no differences between the genotypes according to sex other than for PPI (p’s > 0.05), we combined sexes for biochemical assays experiments from mice. These were analyzed using a 2-way ANOVA (2 genotype × 2 treatment), with a Tukey’s post-hoc test for multiple comparisons to establish differences following a significant interaction. For primary cell culture morphology, averages of all cells from 3 independent replicates were analyzed using a 2-way ANOVA (ASC × LPS) and effects between genotypes were examined with all individual cell values. Primary cell culture gene expression data was analyzed with a 2-way ANOVA (2 genotype × 2 treatment) followed by Tukey’s corrected multiple comparisons to establish differences between treatment groups within each genotype. All HMC3 cell data were analyzed using Univariate ANOVA and significant main effects were followed by a Sidak’s multiple comparisons test. Mice for which PPI was negative were removed from the analyses (n = 1 SVCT2-Tg^BAC−^, mTBI; n = 1 SVCT2-Tg^BAC+^, Sham). Differences among groups were considered significant with a p-value < 0.05.

## 3. Results

### 3.1. SVCT2 transporter expression controls cortical ascorbate levels

Unlike humans, SVCT2^+/+^, SVCT2^+/−^, SVCT2-Tg^BAC−^, and SVCT2-Tg^BAC+^ mice, are all capable of *de novo* ASC synthesis in their liver which can be upregulated under conditions of additional requirement, and so they do not require additional dietary supplementation. Mice were either exposed to a mTBI or sham injury. ASC level did not vary according to sex (p > 0.08), so samples were combined for subsequent analyses. ASC tissue concentration was determined 24 h after blast procedure and there was no effect of blast in either genotype at this time point (p’s > 0.05). ASC depletion in cortical tissue was observed in SVCT2^+/−^ mice which had ~70 % ASC in cortex compared to wild-type littermate controls, SVCT2^+/+^ (Genotype, F_(1, 37)_ = 80.82, p < 0.001, Fig. 1A). As expected, SVCT2-Tg^BAC+^ mice showed 1.5 to 2-fold increase in cortical ASC compared to littermate control SVCT2-Tg^BAC−^ mice (Genotype, F_(1, 30)_ = 32.07, p < 0.001, Fig. 1B). The variability in cortical ASC amount is supported by raw copy number value of the SVCT2 gene in the SVCT2-Tg^BAC+^ mice (p < 0.001, Fig. 1C). Similar levels of depletion and abundance in these mouse models have been established previously (Harrison and May 2009; Harrison et al., 2010; Harrison et al., 2012).

### 3.2. SVCT2^+^/^−^ mTBI mice have increased sensitivity to the prepulse compared to sham controls

**Fig. 2.**
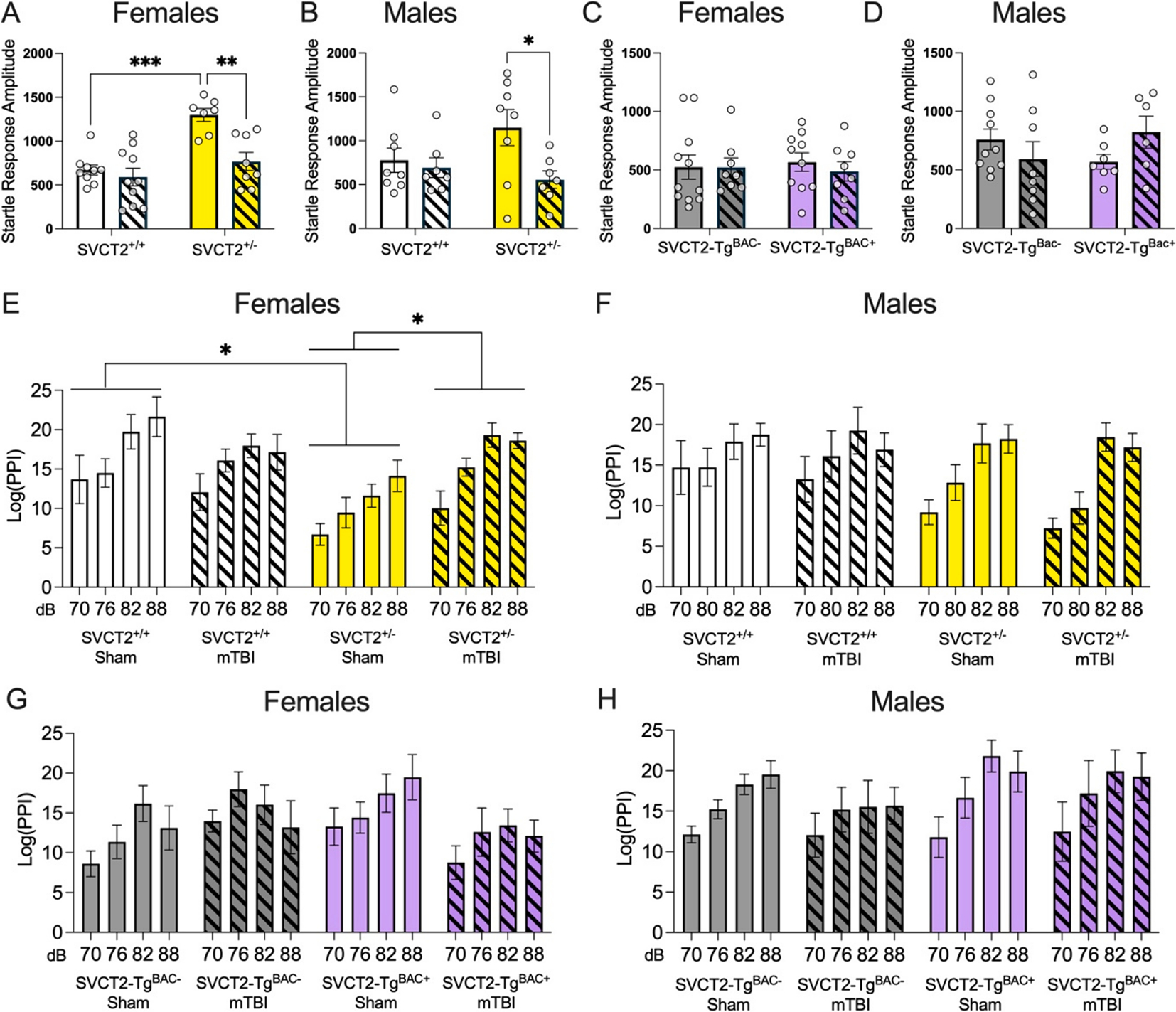
Altered ascorbate transporter expression disrupts prepulse inhibition to the startle response 4 h following mTBI. **(A-B)** Disrupted startle response during control trials 4 h following mTBI in SVCT2^+/+^ and SVCT2^+/−^ female mice **(A)** and male mice **(B)**. **(C-D)** No differences to startle response amplitude during control trials 4 h following mTBI in SVCT2-Tg^BAC−^ and SVCT2-Tg^BAC+^ female **(C)** or male **(D)** mice. **(E-F)** PPI response is disrupted for all prepulse decibel strengths in SVCT2^+/+^ and SVCT2^+/−^ female **(E)** and male **(F)** mice. **(G-H)** No changes in PPI response for any prepulse decibel strengths in SVCT2-Tg^BAC−^ and SVCT2-Tg^BAC+^ female **(G)** or male **(H)** mice 4 h following mTBI. Data are presented as ± S.E.M with n = 7–10 males or females per group. *p < 0.05, ** p < 0.01, ***p < 0.001 significantly different as marked, pairwise comparisons following significant ANOVA.

Previous research has found long term disruptions to behavior and cognition following mTBI in wild type mice (Mouzon et al., 2018; Eyolfson et al., 2020). Prepulse inhibition of the startle response (PPI) is a behavioral task that is sensitive to changes in sensorimotor gating across multiple neurological conditions including TBI (Parwani et al., 2000; Kohl et al., 2013; Brooks et al., 2016). The PPI task was chosen to measure TBI-related damage within the acute time period after injury (24 h). Mice that received a mTBI tend to show decreased motivation and activity (e.g. huddling in the corner of the cage) and the PPI task can be conducted independent of animal movement or motivation. We therefore determined whether a SVCT2 heterozygous knockdown would affect acute sensorimotor gating at 4 h following mTBI, and whether SVCT2 overexpression is protective against mTBI. In both male and female mice, we observed an increased startle response to the 120 dB tone in sham injured SVCT2^+/−^ mice combined with a large attenuation of the startle response in mTBI injured SVCT2^+/−^ animals compared to sham injured controls (Females: *Genotype*, F_(1,62)_ = 14.45p < 0.001, *mTBI*, F_(1,62)_ = 11.15p = 0.001, *Genotype × mTBI,* F_(1,62)_ = 7.34p = 0.009, Fig. 2A). A similar pattern of effects was observed in male mice with the largest response to mTBI observed in the SVCT2^+/−^ mice (*TBI*, F_(1,54)_ = 4.67, p = 0.035, Fig. 2B). There were no changes to raw startle response in SVCT2-Tg^BAC−^ or SVCT2-Tg^BAC+^ female or male mice due to genotype or mTBI (p’s > 0.07, Fig. 2C and D).

PPI was calculated relative to baseline startle and log transformed to better assess relative changes among the groups. In female mice, an overall decrease in PPI in SVCT2^+/−^ mice was again driven by the sham injured SVCT2^+/−^ animals (genotype F_(1,30)_ = 4.38, p = 0.045, genotype × injury F_(1,30)_ = 4.33, p = 0.046, Fig. 2E). In males, decreased PPI was observed in both sham and mTBI injured SVCT2^+/−^ mice (dB × SVCT2 genotype F_(3,81)_ = 2.95, p = 0.04, Fig. 2F) with greater differences observed at the lowest prepulse which represents the most challenging stimulus to differentiate (76 dB, p = 0.03).

In SVCT2-Tg^BAC−^ female mice mTBI was associated with increased PPI whereas in SVCT2-Tg^BAC+^ mTBI led to decreased PPI (Fig. 2G, *Genotype × mTBI*, F_(1,31)_ = 4.13, p = 0.05), however, no specific differences were detected following post hoc Sidak pairwise comparisons. No changes in PPI were observed in SVCT2-Tg^BAC−^ or SVCT2-Tg^BAC+^ males due to TBI or genotype (p’s > 0.33, Fig. 2H). These data suggest that decreased SVCT2 and concomitant decreases in ASC in the brain expression can negatively impact startle response and sensorimotor gating following mTBI while overexpression of SVCT2 prevented similar changes in male mice, and at least partially attenuated changes in female mice.

### 3.3. mTBI causes mild changes to microglial morphology

Although the behavioral findings suggest ASC and SVCT2 may be involved in the neural response to PPI it is not possible to differentiate which cell types are implicated from those data. Microglia are the immune modulators of the central nervous system, and have altered inflammatory profile following TBI (Chiu et al., 2016; Donat et al., 2017; Ritzel et al., 2020). Under normal environmental conditions microglia have highly ramified processes, whereas under an inflammatory state microglia rapidly shift to a more amoeboid morphology with fewer processes (Nayaket al., 2014; Donat et al., 2017; Morrison et al., 2017). ASC may mediate microglial morphology via expression of SVCT2 and other neuroinflammatory genes following a pro-inflammatory stimulus (Portugal et al., 2017; Portugal et al., 2017; Portugal, 2024).

**Fig. 3.**
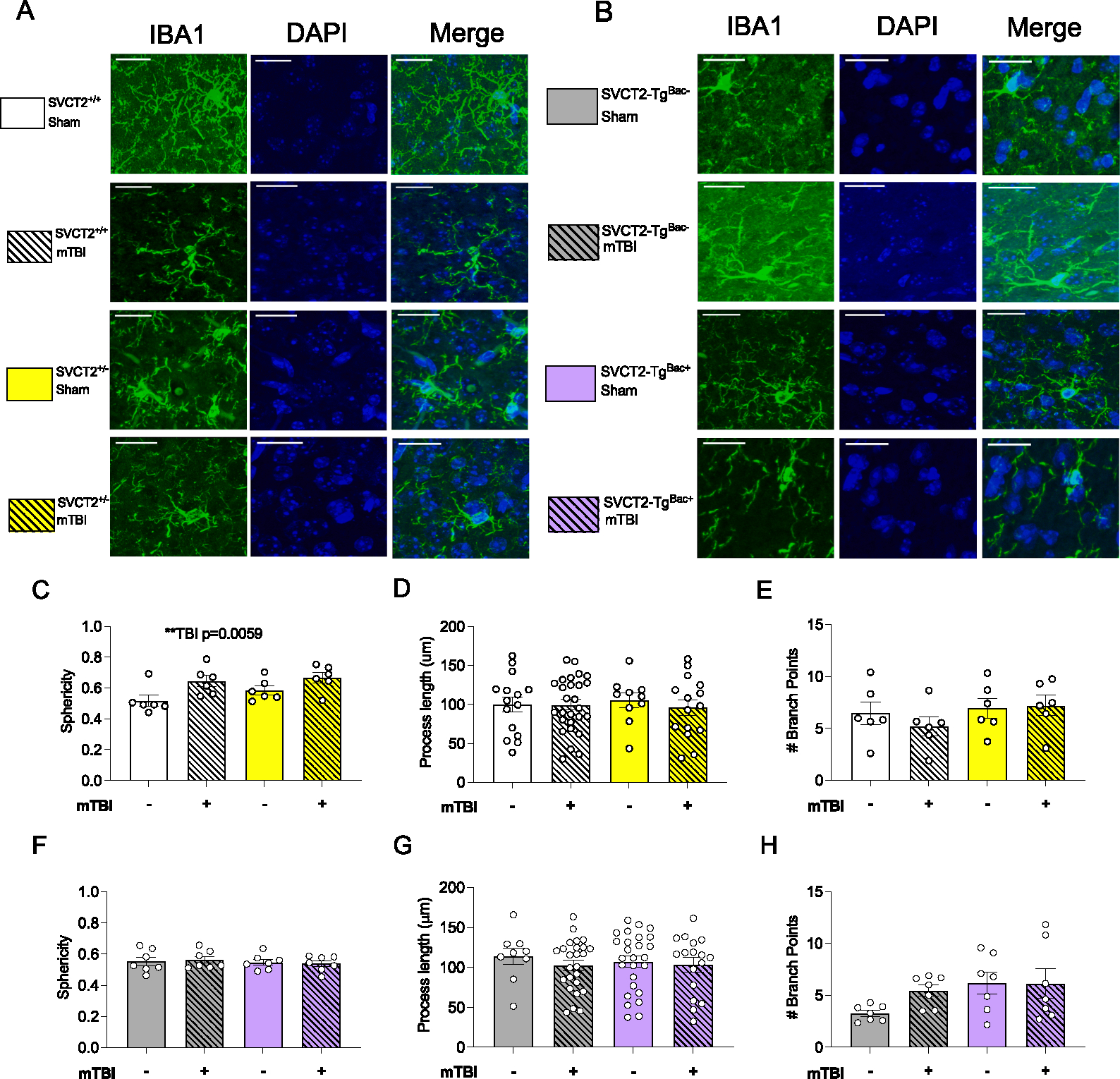
Mtbi causes minimal morphological changes in microglia24 h after injury in SVCT2^+/−^ mice. **(A-B)** Representative images of microglia stained with IBA-1 in **(A)** SVCT2^+/+^ and SVCT2^+/−^ and **(B)** SVCT2-Tg^BAC−^ and SVCT2-Tg^BAC+^ frontal cortex. **(C)** Average Sphericity **(D)** length of processes, and **(E**) number of branching points of microglia from SVCT2^+/+^ and SVCT2^+/−^ mice. **(F)** Average Sphericity **(G)** Length of processes and **(H)** Number of branching points per process of microglia from SVCT2-Tg^BAC−^ and SVCT2-Tg^BAC+^ mice. Data are presented as ± S.E.M with main effects marked on individual graphs. Data shown are averages of at least n = 4–6 microglia in the frontal cortex per mouse, with n = 3–5 males and female brains used per group. Scale bar represents 20 μm.

To investigate the role of SVCT2 in microglial morphology in mTBI, at 24 h post mTBI, a subset of mice was euthanized, and the front half of their brains was fixed, sliced and stained with IBA-1 prior to analysis of microglial morphology in the frontal cortex over which the mTBI was directed. Microglia in the frontal cortex were visualized using confocal microscopy and analyzed using IMARIS software to determine more detailed morphological features (Fig. 3A and B, representative images). mTBI increased sphericity in both SVCT2^+/+^ and SVCT2^+/−^ mice compared to sham-injured controls (Fig. 3C, mTBI, F_(1,20)_ = 9.49, p = 0.006). Neither mTBI nor genotype impacted the number of cellular processes per microglia nor number of branching points per process (p’s > 0.21, Fig. 3D and E). The effects were mild in these genotypes and mTBI did not further exacerbate microglial changes in SVCT2^+/−^ mice compared to their SVCT2^+/+^ littermate controls. No differences in sphericity, process length or branching points were observed in the SVCT2-Tg^BAC−^ and SVCT2-Tg^BAC+^ mice (p’s > 0.07, Fig. 3F–H). These data suggest that even with a mild TBI some evidence of microglial reactivity can be detected up to 24 h later. The effect was more visible in the SVCT2^+/−^ mouse line whereas mice bred within the SVCT2-Tg^BAC+^ line may have been protected suggesting that there may be some differences even between the wild-type controls within the two lines. Potential differences in inflammatory response due to developmental availability of ASC were not explicitly tested in this experiment.

### 3.4. SVCT2 heterozygosity increased NF-κB pathway expression changes

**Fig. 4.**
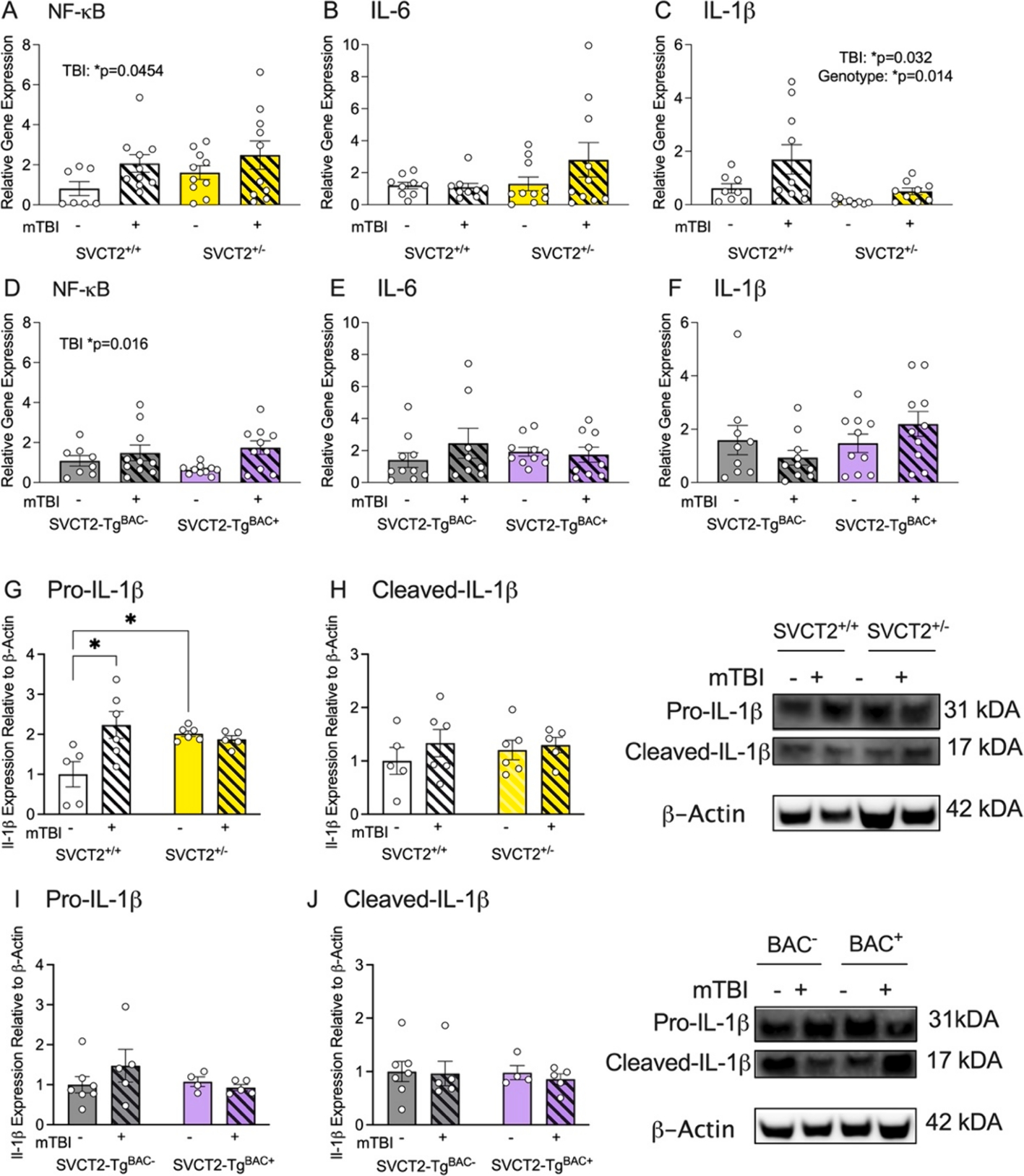
Depleted SVCT2 expression disrupts NF-κB pathway signaling. **(A-C)** NF-κB and proinflammatory cytokine (IL-6, IL-1β) signaling in SVCT2^+/+^ and SVCT2^+/−^ mice 24 h after mTBI. **(D-F)** NF-κB and proinflammatory cytokine (IL-6, IL-1β) signaling in SVCT2-Tg^BAC−^ and SVCT2-Tg^BAC+^ mice 24 h after mTBI. **(G-H)** Pro- and cleaved-IL-1β protein in SVCT2^+/+^ and SVCT2^+/−^ mice. **(I-J)** Pro- and cleaved-IL-1β protein in SVCT2-Tg^BAC−^ and SVCT2-Tg^BAC+^ mice. Data are presented as ± S.E.M, main effects are noted on individual graphs, n = 7–10 total mice (4–6 males or females) per group for mRNA. 2–4 males or females per group for protein expression.

ASC has been shown to prevent NF-κB translocation to the nucleus in human neuroblastoma cells (Son et al., 2004). Thus, we next determined whether there was a similar direct effect of ASC transporters on microglial activation and the NF-κB pathway. We analyzed the expression levels of the genes coding for transcription factor NF-κB and proinflammatory cytokines IL-6 and IL-1β in the frontal cortex 24 h post mTBI. We found an increase in NF-κB mRNA expression in SVCT2^+/+^ and SVCT2^+/−^ mice following mTBI (Fig. 4A, *mTBI*, F_(1,33)_ = 4.33, p = 0.045). There were no significant differences in IL-6 expression among the groups (Fig. 4B). There was a slight increase in expression of the downstream proinflammatory cytokine Il-1β gene following mTBI in both the SVCT2^+/+^ and SVCT2^+/−^ mice although this response was blunted in the SVCT2^+/−^ mice (Fig. 4C, m*TBI*, F_(1,32)_ = 5.044, p = 0.032, genotype, F_(1,32)_ = 6.7, p = 0.014). The same increase in NF-κB expression following mTBI was observed in the SVCT2-Tg^BAC−^ and SVCT2-Tg^BAC+^ mice, with no additional differences due to genotype (Fig. 4D, m*TBI*, F_(1,34)_ = 6.34, p = 0.017) and no additional other differences in IL-6 or IL-1β gene expression in any group (Fig. 4E and F).

Next we looked expression of both pro-IL-1β and cleaved-IL-1β proteins to identify whether caspase activity and inflammasome involvement had occurred. We observed an increase in pro-IL-1β following mTBI in SVCT2^+/+^ mice, while elevated pro-IL-1β in SVCT2^+/−^ shams was not further increased with mTBI (Fig. 4G, *Interaction*, F_(1,18)_ = 8.0, p = 0.011). There were no significant changes to cleaved-IL-1β protein in SVCT2^+/+^ and SVCT2^+/−^ cortex (Fig. 4H, p > 0.34). There were no changes in the SVCT2-Tg^BAC−^ or ^BAC+^ pro-IL-1β or cleaved-IL-1β (Fig. 4I and J, p’s > 0.23). These data support the earlier observation of altered microglial morphology and suggest that even a mild TBI is sufficient to induce changes in microglia, such as increased NF-κB and IL-1β gene expression and protein changes, can be detected 24 h later. As before, these changes were more evident in the SVCT2^+/−^ mouse line.

### 3.5. ASC attenuates morphology changes in primary microglial cell culture due to LPS

To better understand the impact of ASC and SVCT2 on microglial function we then directly probed changes in isolated microglial to determine whether the effects of an inflammatory stimulus depend on SVCT2 genotype for microglia. Microglia were isolated from pups (P0-P2) from each genotype and treated with 100 ng/mL of LPS with and without ASC for 24 h, then fixed and stained with IBA-1 prior to imaging them for changes in morphology (Supplemental Fig. 1).

**Fig. 5.**
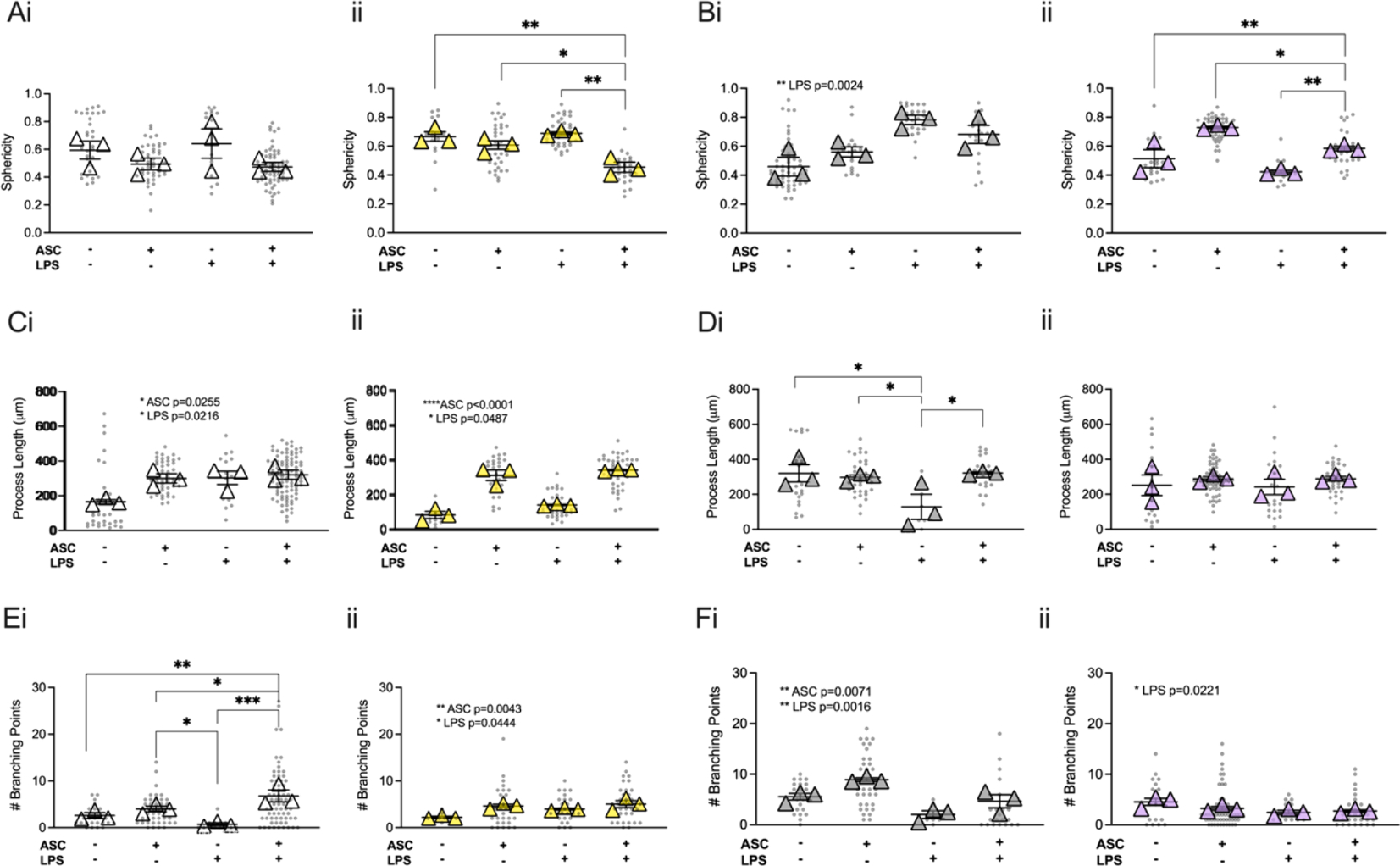
ASC is protective against primary microglial morphology changes following LPS treatment. **(A-B)** Sphericity of microglia in SVCT2^+/+^ and SVCT2^+/−^ **(Ai, Aii),** and SVCT2-Tg^BAC−^, SVCT2-Tg^BAC+^ cells **(Bi, Bii). (C-D)** Length of processes of microglia in SVCT2^+/+^ and SVCT2^+/−^ **(Ci, Cii),** and SVCT2-Tg^BAC−^, SVCT2-Tg^BAC+^ cells **(Di, Dii). (E-F)** Number of process branching points of microglia in SVCT2^+/+^ and SVCT2^+/−^ **(Ei, Eii)**, and SVCT2-Tg^BAC−^, SVCT2-Tg^BAC+^ cells **(Fi, Fii).** Data are presented as ± S.E.M with main effects noted on individual graphs. *p < 0.05, **p < 0.01, ***p < 0.001, ****p < 0.0001. Data are presented as ± S.E.M main effects are noted on individual graphs. Data shown are average values of all cells (grey dots) measured during 3 independent experimental replicates (n = 3, triangles).

Overall, greater sphericity was observed in cells from SVCT2^+/−^ mice than those from SVCT2^+/+^ mice when comparing individual cells (*Genotype*, F_(1,267)_ = 3.96p = 0.048, Fig. 5Aii) suggesting either that decreased SVCT2^+/−^ expression led to more amoeboid phenotype at baseline, or they were more impacted by the potential stress of being in culture. Significant differences among the treatment groups were, therefore, analyzed separately for each genotype (Fig. 5Ai, 5Aii *Treatment*, p < 0.001). In cells from SVCT2^+/+^ mice addition of ASC did not alter sphericity compared to untreated cells regardless of addition of LPS (p’s > 0.08). In SVCT2^+/−^ cells a protective effect of ASC was observed when it was added in combination with LPS. No significant impact of added ASC was observed in the absence of LPS suggesting the response may have been limited by decreased expression of SVCT2 and that benefits of added ASC were only therefore observed in the greater stress conditions (Fig. 5Aii *Interaction* F_(1,8)_ = 9.37, p = 0.016). SVCT2-Tg^BAC+^ cells exhibited lower sphericity than cells from SVCT2-Tg^BAC−^ mice overall suggesting a less inflammatory phenotype although this effect depended on treatment condition (Fig. 5Bi, Bii, *Genotype* F_(1,214)_ = 9.51, p = 0.002; *Treatment* F_(3,214)_ = 16.37, p < 0.001; *Interaction*, F_(3,214)_ = 33.04, p < 0.001). As above, data were analyzed individually for each genotype, and were collapsed across experiments rather than analyzing individual cells. In SVCT2-Tg^BAC−^ microglia, exposure to LPS led to increased sphericity that was not significantly attenuated by ASC (Fig. 5Bi, *LPS* F_(1,8)_ = 19.09, p = 0.002), and ASC alone did not impact sphericity (p > 0.98). Surprisingly, in SVCT2-Tg^BAC+^ cells sphericity was increased in cells treated with ASC and ASC with LPS, but not LPS alone (Fig. 5Bii, *ASC* F_(1,8)_ = 33.39, p < 0.001, *LPS* F_(1,8)_ = 12.88, p = 0.007) suggesting that increasing ASC above physiological levels may not always be protective.

Average length of processes was shorter in microglia from SVCT2^+/−^ mice compared to SVCT2^+/+^ controls although length was strongly dependent on treatment condition (Fig. 5Ci, Cii, *Genotype* F_(1,286)_ = 12.87, *Treatment* F_(3,286)_ = 29.84, p < 0.0001, *Interaction*, F_(3,286)_ = 8.3, p < 0.001). When data were analyzed separately within each genotype and cell data were collapsed across experiments all SVCT2^+/+^ microglia groups treated with ASC or LPS had longer processes than the media alone group (Fig. 5Ci, *ASC*, F_(1,8)_ = 7.51p = 0.03, *LPS* F_(1,8)_ = 8.10p = 0.02). In SVCT2^+/−^ expressing microglia cells treated with ASC had longer processes then those with media or LPS alone (Fig. Cii, *ASC*, F_(1,8)_ = 132.3, p < 0.001), and shorter processes in LPS treated cells overall (Fig. 5Cii, *LPS* F_(1,8)_ = 5.4, p = 0.049), though there was no interaction effect (p = 0.49). In SVCT2-Tg^BAC−^ cells, LPS treated cells had significantly shorter processes than media or ASC-treated cells, and the decreased length was rescued by ASC (Fig. 5Di, *Interaction*, F_(1,8)_ = 5.98, p = 0.04). There were no significant differences in process length according to ASC or LPS treatment in SVCT2-Tg^BAC+^ cells (Fig. 5Dii, p’s > 0.3).

The number of branch points of processes was used as an additional marker of morphological complexity with lower number of branch points indicating a more reactive cell or more amoeboid shape. Although there was no overall difference according to genotype, SVCT2^+/+^ cells were more responsive to the impact of LPS and ASC treatment than cells from SVCT2^+/−^ mice (Fig. 5Ei, Eii, *Treatment*, F_(3,247)_ = 8.89, p < 0.001, *Interaction*, F_(3,247)_ = 5.59, p = 0.001,). In SVCT2^+/+^ cells, surprisingly ASC and LPS treated cells had the highest number of branch points though LPS alone had significantly fewer compared to ASC treated cells (Fig. 5Ei). In contrast SVCT2^+/−^ cells had significantly more branching points in ASC treated cells regardless of LPS treatment (Fig. 5Eii). Overall the number of branch points was fewer in SVCT2-Tg^BAC+^ microglia than control mice, however, this effect was dependent on treatment condition (*Interaction*, F_(3,194)_ = 5.88, p < 0.001, *Genotype*, F_(1,194)_ = 5.83, p = 0.02, *Treatment*, F_(3,194)_ = 7.65, p < 0.001, Fig. 5F). There were no differences in number of branch points due to treatment in SVCT2-Tg^BAC+^ microglia. ASC treatment was associated with increased number of branch points in SVCT2-Tg^BAC−^ microglia, and although branch points were lowest in the LPS alone treated group this effect was not significantly attenuated by ASC (p > 0.05). Together these data suggest that treatment with ASC was protective against LPS in maintaining microglial homeostatic morphology under some conditions. Furthermore, the data suggest that changes are not driven solely by ASC availability, but also by amount of transporter since in general we observed increased sensitivity to challenge and rescue in SVCT2^+/−^ cells and a decreased impact of treatments in SVCT2-Tg^BAC+^ cells.

### 3.6. SVCT2 attenuates NF-κB pathway activation following to LPS treatment in primary cells

**Fig. 6.**
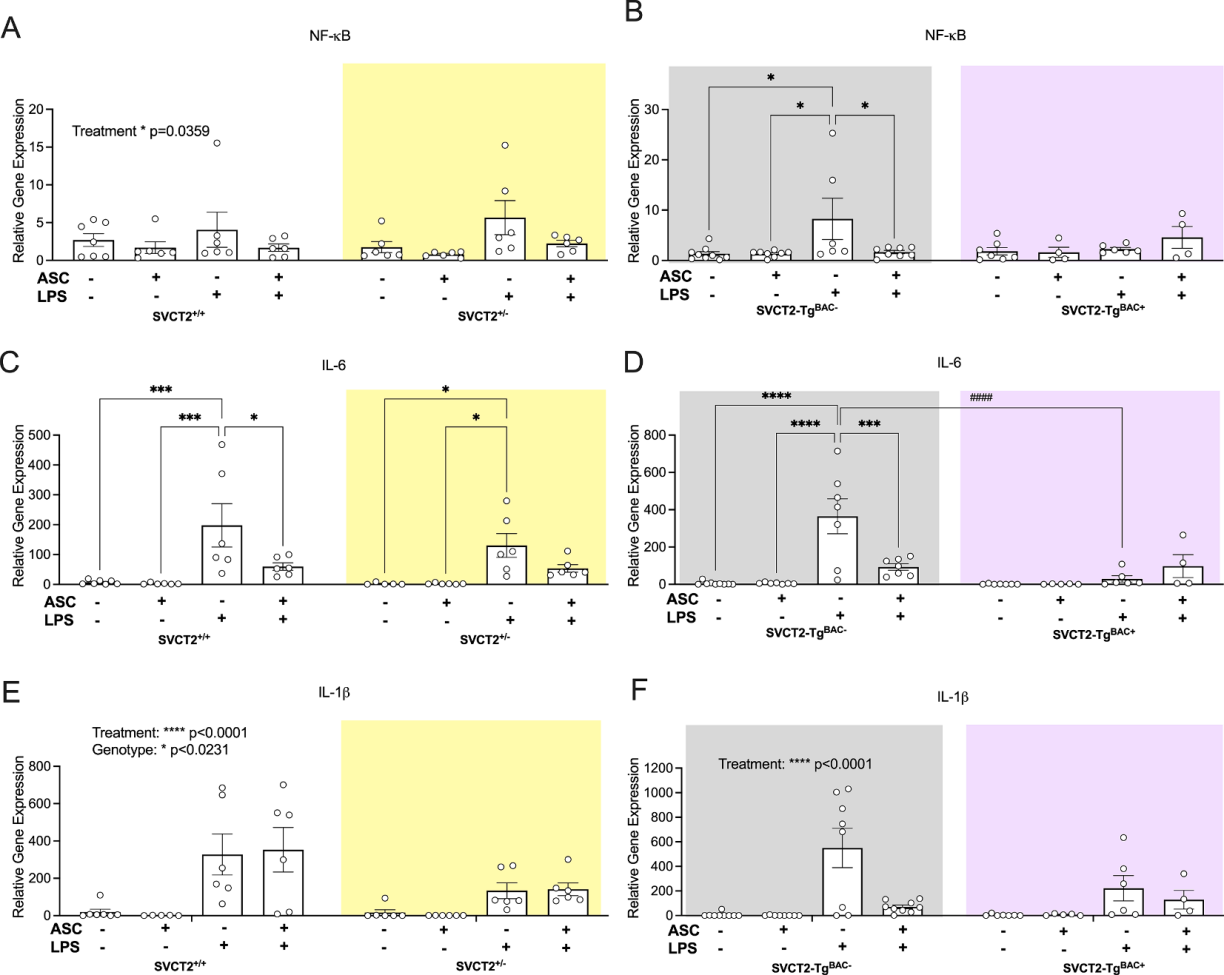
Microglia with depleted SVCT2 expression have disrupted NF-κB pathway signaling, and overexpression is protected following LPS treatment. **(A-B)** NF-κB mRNA expression in SVCT2^+/+^ and SVCT2^+/−^ microglia **(A)** and SVCT2-Tg^BAC−^ and SVCT2-Tg^BAC+^ microglia **(B)**. **(C-D)** IL-6 signaling in SVCT2^+/+^ and SVCT2^+/−^ microglia **(C)** and SVCT2-Tg^BAC−^ and SVCT2-Tg^BAC+^ microglia **(D)**. **(E-F)** IL-1β signaling in SVCT2^+/+^ and SVCT2^+/−^ microglia **(E)**, and SVCT2-Tg^BAC−^ and SVCT2-Tg^BAC+^ microglia **(F)** 24 h after mTBI. Data are presented as ± S.E.M with main effects noted above individual graphs. *p < 0.05, **p < 0.01, ***p < 0.001, ****p < 0.0001 effects within genotype, ^#^p < 0.05, ^###^p < 0.001, ^####^p < 0.0001 effects between genotypes. N = 5–9 independent replicates treated per group.

To better understand what may be driving the morphological changes and reactivity we also studied NF-κB pathway activation within the different genotypes following LPS treatment. NF-κB gene expression did not change due to LPS or ASC treatment in SVCT2^+/+^ cells, however, SVCT2^+/−^ cells were sensitive to LPS treatment (*Treatment*, F_(3,41)_ = 3.13, p = 0.036, Fig. 6A). A significant increase in NF-κB expression (fold change) was seen in SVCT2^+/−^ cells following LPS treatment but not when cells were also treated with ASC. Similarly, SVCT2-Tg^BAC−^ cells also appeared more sensitive to LPS treatment than SVCT2-Tg^BAC+^ cells (*Treatment* F_(3,44)_ = 2.74, p = 0.05 *Interaction*, F_(3,44)_ = 2.99, p = 0.04, Fig. 6B). Increased NF-κB expression following LPS treatment (p’s < 0.01) was attenuated by ASC in the wild-type cells (p < 0.05), whereas SVCT2-Tg^BAC+^ cells, were resistant to both LPS and ASC treatment (p’s > 0.05). Il-6 was significantly increased in both SVCT2^+/+^ and SVCT2^+/−^ cells following LPS treatment, (Fig. 6C, *Treatment*, F_(3,40)_ = 12.73, p < 0.001). The impact of LPS was attenuated in both genotypes with ASC treatment although the difference was only significant in SVCT2^+/+^ cells (p < 0.05; SVCT2^+/−^ p = 0.28). A similar trend was observed in SVCT2-Tg^BAC−^ cells (*Genotype*, F_(1,44)_ = 8.94, p = 0.005, *Treatment*, F_(3,44)_ = 11.34p < 0.0001, *Interaction*, F_(3,44)_ = 9.03, p < 0.001 Fig. 6D,) in which IL-6 expression was significantly increased in response to LPS (p’s *<* 0.001) and this was attenuated with ASC treatment (p *<* 0.001). No change due to LPS or ASC treatment was observed in the SVCT2-Tg^BAC+^ cells. Finally, IL-1β mRNA expression increased following LPS treatment in SVCT2^+/+^ cells (p’s < 0.01) and this effect was not attenuated by ASC treatment (p = 0.99). Interestingly SVCT2^+/−^ cells showed an attenuated response to LPS in IL-1β mRNA expression and neither were they impacted by ASC treatment (p’s > 0.38) (*Genotype*, F_(1,40)_ = 5.59p = 0.023, *Treatment*, F_(3,40)_ = 9.36p < 0.001, Fig. 6E,). Inflammatory response was again lower in SVCT2-Tg^BAC+^ cells compared to controls although this effect did not reach significance (Fig. 6F, *Treatment*, F_(3,48)_ = 11.72, p < 0.001, *Interaction,* F_(3,48)_ = 2.68, p = 0.0057). In SVCT2-Tg^BAC−^ cells the expected increase in LPS treatment (p’s < 0.001) was again attenuated with ASC (p < 0.001). No changes were observed in SVCT2-Tg^BAC+^ cells between treatments (p’s > 0.20). The lack of changes to proinflammatory gene expression suggest expression of SVCT2, regardless of supplementation of ASC, is required for appropriate microglial activation.

### 3.7. Antioxidants protect HMC3 cells from proinflammatory cytokine signaling and cell death following LPS

**Fig. 7.**
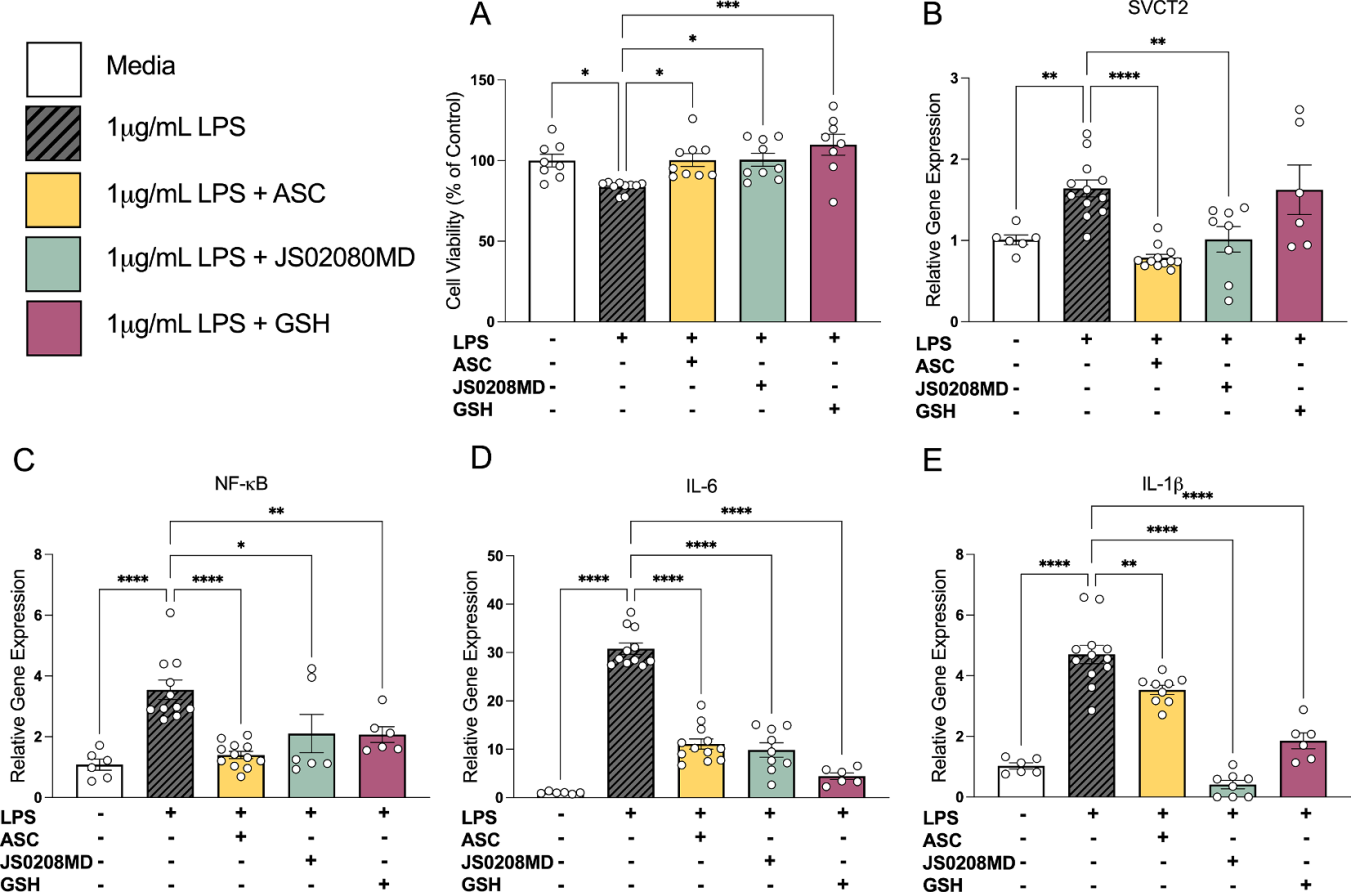
Attenuation of neuroinflammatory gene expression is not specific to ASC in HMC3 cells. **(A)** 1 μg/mL LPS decreases cell viability, which is rescued by ASC, JS0208MD, and GSH. **(B)** SVCT2 mRNA expression is increased with 1 μg/mL LPS, which is attenuated by treatment with ASC or JS0208MD, but not GSH. Relative proinflammatory cytokine mRNA of NF-κB **(D),** IL-6 **(E),** and IL-1β **(F)** is increased with 1ug/mL LPS, and attenuated by ASC, JS0208MD and GSH. n = 6–12 independent cultures. Data are presented as ± S.E.M *p < 0.05, **p < 0.01, ***p < 0.001, ****p < 0.0001 (One-way ANOVA), n = 6–12 independent replicates per group.

To further clarify the potential protective roles for SVCT2-mediated and ASC uptake on the microglial response we treated HMC3 cells with LPS (1 μg/mL) with or without one of the following antioxidants: ASC, JS0208MD, or GSH. A higher dose of LPS was used in this experiment because HMC3 cells are less sensitive to LPS than primary cells (Dello Russo et al., 2018). JS0208MD is an experimental botanical extract that contains high ASC concentration as well as minerals and other polyphenols. The concentration of JS0208MD that was used was matched to the ASC levels used in cells treated with ASC alone. Cells treated with LPS had significantly decreased cell viability, which was prevented following co-treatment with ASC, JS0208MD, or GSH (Fig. 7A, *Treatment*, F_(4,39)_ = 5.77, p = 0.001). SVCT2 mRNA expression was increased with LPS treatment and this response was prevented by treatment with ASC or JS0208MD, but not GSH (*Treatment*, F_(4,39)_ = 9.74, p < 0.001 Fig. 7B). NF-κB expression increased with LPS treatment and was also attenuated by treatment with all antioxidant compounds (*Treatment*, F_(4,36)_ = 11.23, p < 0.001 Fig. 7C). Similar patterns were seen in IL-6 and IL-1β expression (IL-6: *Treatment*, F(_(4,39)_ = 101.1, p < 0.0001 Fig. 7D, IL-1β; *Treatment*, F_(4,39)_ = 60.79, p < 0.001 Fig. 7E). Although all antioxidants attenuated cytotoxicity and LPS-induced increases in NF-κB, IL-6 and IL-1β expression, the data suggest that they have differential efficacy in attenuating some outcomes in ROS-mediated pathways of inflammation.

## 4. Discussion

In the current study, we sought to elucidate how SVCT2 expression may impact microglial response following an inflammatory trigger. ASC and its transporter, SVCT2, are proposed to play a role in mediating microglial shift between homeostatic and activated states (Portugal et al., 2017; Portugal et al., 2017; Portugal, 2024). Previous studies were performed using *in vitro* techniques, and we therefore sought to replicate and extend the findings using *in vivo* SVCT2 partial knockout and overexpression rodent models and primary cell lines. We used established models of mTBI and LPS-induced neuroinflammation, in which others have previously determined a role for microglial-mediated neuroinflammation (Heldt et al., 2014; He et al., 2021). Independent littermate controls were used for each mouse line due to known differences in development with altered ASC levels (Harrison et al., 2010; Pastor et al., 2013; Salazar et al., 2021) allowing us to account for any developmental differences and make more direct observation of effects relevant to SVCT2 expression rather than other potential confounding factors. These models were reflective of global SVCT2 changes *in vivo*. In order to fully address the role of SVCT2 on microglia specifically, conditional or cell specific knockout models or overexpression models should be used. Since the majority of rodent models cannot fully recapitulate what is seen in the human mTBI situation due to their ability to increase synthesis of ASC, future studies may also utilize the gulo^−/−^ mouse model to account for this.

PPI is a commonly utilized translationally-relevant measure that is useful in the assessment of behavioral changes in a number of neurological disorders (Takahashi et al., 2011; Kohl et al., 2013). The task requires subconscious attentional processes to mediate sensorimotor gating in the presence of auditory stimuli. It directly measures the ability to filter out unnecessary stimuli in the environment, which is a key feature of selective attention (Gómez-Nieto et. al., 2020). PPI has been associated with a number of different regions within the brain along the cortico-striatal-pallido-thalamic network (Swerdlow et al., 1999; Fendt et al., 2001; Geyer et al., 2001). Since it is an attentional task, examining the frontal cortex is still an important part of the neural system required, but potential future directions should examine all of these areas involved to determine where along the pathway is mTBI interfering. This is likely particularly important in cases where the injury is more severe and may also include rotational-acceleration injuries (e.g. axonal shearing). This PPI task relies on the ability to hear the auditory stimuli and it has been known for decades that there is an age-related hearing loss that occurs in the inbred C57BL/6J mice (Mikaelian et al., 1974) which is the background strain for both of the SVCT2 mouse lines. Hearing is relatively normal in most C57BL/J6 mice at 3 months of age and progresses as animals age (Mikaelian et al., 1974; Ison et al., 2007). Nevertheless, we controlled for potential hearing loss by removing animals which did not startle at all (negative or zero value) from PPI and subsequent analyses. Greater behavioral differences were seen in SVCT2^+/−^ mice in which we saw a larger magnitude of decreased startle in mTBI mice compared to sham injured mice. Additionally, female SVCT2^+/−^ mice had a higher startle response, and decreased prepulse inhibition compared to SVCT2^+/+^ controls. This decreased inhibition by the prepulse may reflect diminished arousal capability or responsiveness during prepulse trials. The attenuation of the increase in startle response in SVCT2^+/−^ mice that received a mTBI could be attributed to increased sensitivity to the prepulse and is not that mTBI was protective from ASC-induced changes to the startle response. Congruent with our predictions, neither male or female SVCT2-Tg^BAC+^ had significantly different responses compared to controls. This suggests these mice may be protected from acute cognitive changes following mTBI. Altered PPI has been reported in other mTBI models and has been shown to persist following mTBI, however, these results are the first to our knowledge of this effect being reported in such an acute model (Pang et al., 2015; Sinha et al., 2017). It is possible that the size difference (possibly in the skull thickness or muscle tissue) in males allows them to withstand a mTBI better than females, although studies in humans and rat models have also revealed sex differences in PPI (Lehmann, 1999; Swerdlow et al., 1999; Kumari et al., 2003, 2008).

Microglia respond rapidly follow mTBI with changes reported within 24 h following injury (Morganti et al., 2015; Donat et al., 2017). Although the injury used in this study was mild, we predicted the combined effects of SVCT2 expression and injury would elicit subtle changes including prolonged inflammatory response in the ASC deficient animals. In other models, with more severe injuries significant neuroinflammatory changes occurred by 24 h post injury (Donat et al., 2017; Witcher et al., 2021). We observed a significant increase in sphericity of microglia in SVCT2^+/−^ mice with no further changes to any other morphological features, with no changes occurring between the SVCT2-Tg^BAC−^ or SVCT2-Tg^BAC+^ microglia. Similar results using the same blast model, failed to observe gliosis by immunohistochemical staining, although microglial morphology was not considered (Heldt et al., 2014). Therefore, while a more severe injury or shorter post injury time-points may have better highlighted the potential impact of ASC and SVCT2 on microglial activation *in vivo*, we instead utilized primary cultured microglia from each mouse line with LPS as an inflammatory trigger (Gessi et al., 2016; Santa-Cecília et al., 2016). Activation of the NF-κB transcription pathway with inflammasome activation can lead to subsequent cleavage of Il-1β by caspase-1 and further secretion of inflammatory factors (Martín-Sánchez et al., 2016). The NF-κB signaling pathway can be directly impacted by ASC via two mechanisms. First, by inhibiting oxidative free radical signaling initiators via ASC accumulation within the cell, and through the oxidized form of ASC, DHA directly binding and preventing the phosphorylation and degradation of IκB kinase (IKK) thus allowing the NF-κB complex to translocate to the nucleus and upregulate proinflammatory genes (Cárcamo et al., 2004; Engelmann et al., 2014; Marino et al., 2023). SVCT2-Tg^BAC−^ and SVCT2-Tg^BAC+^ cortical tissue exhibited the least gene expression changes and subsequent protein expression of Il-1β relating to this pathway. We hypothesized that the lack of differences in response to injury in these mice may be due to some conferred developmental protection from having one parent with an overexpression of SVCT2, including in placenta, even if the mice themselves do not have significant overexpression.

SVCT2^+/−^ cells grown in media alone had a more spherical shape at baseline compared to SVCT2^+/+^, SVCT2-Tg^BAC−^ and SVCT2-Tg^BAC+^ cells indicating differences in activation state. Furthermore, in several cases the inflammatory response induced by LPS was attenuated by ASC co-treatment. Similarly, SVCT2^+/−^ cells had an increase in mRNA expression in NF-κB, IL-6 and IL-1β, and ASC co-treatment with LPS did not correct signaling changes to control levels, presumably because transport of ASC was restricted even when it was available in the media. In contrast, ASC cotreatment with LPS in the SVCT2^+/+^ cells attenuated IL-6 signaling. SVCT2-TG^BAC+^ microglia also showed fewer changes in morphology due to LPS treatment than SVCT2-TG^BAC−^ control cells. Therefore, the increased disruption to this pathway in SVCT2^+/−^ and protection in SVCT2-Tg^BAC+^ cells suggests a potential mechanism mediating neuroinflammatory changes in microglia. The differences between NF-κB, IL-6 and IL-1β expression *in vivo* among the genotypes could be explained by differences in cell types. In addition to microglia, SVCT2 is expressed in multiple cell types including neurons, oligodendrocytes, and endothelial cells, though the highest expression is thought to be in neurons (Mun et al., 2006). Data for these experiments was obtained from whole tissue samples in the area of the cortex directly impacted by the blast injury. We did not isolate microglia to look at cell type specific changes which explains why the results are more distinct in microglial specific *in vitro* experiments compared to *in vivo* conditions. For future studies, using cell sorting and immunofluorescent staining for NF-κB in the nucleus would reflect more accurately NF-κB translocation and subsequent activation.

Although the impact of altered SVCT2 in the *in vivo* mouse models was small due to the mild injury and lack of a single focal point of damage it is striking that we observed similar changes in expression when extending the findings in *in vitro* models. This suggests that SVCT2 is required in order to maintain redox balance, and when expression is disrupted, they are primed to be activated. Thus, even as the results in the mice and primary cells supported our hypothesis that altered SVCT2 expression would disrupt microglia through a direct impact on NF-κB signaling, it was important to further test whether these changes were specific to ASC transport or whether other antioxidants may be as effective. Cell viability and NF-κB pathway signaling were attenuated by ASC, by ASC in combination with a complex plant-derived and antioxidant rich botanical, JS0208MD, and by GSH. While this suggests that a general antioxidant-rich environment will promote microglial homeostasis, we also showed that SVCT2 expression in response to LPS was only prevented by ASC-containing treatments. Further, ASC, JS0208MD, and GSH were not equally effective in each outcome suggesting a synergistic effect of ASC combined with other antioxidant compounds. GSH is ubiquitous throughout most cells in the central nervous system. Although the concentration of GSH is normally moderate in ramified, homeostatic microglia, high concentrations are detected in microglia under oxidative stress (Chatterjee et al., 1999; Hirrlinger et al., 2000). GSH is synthesized directly from cysteine and is therefore freely available in almost all cell types and does not depend on a transporter system (Lu, 2013). GSH also plays an important role in recycling of ASC from the oxidized form of dehydroascorbic acid (May et al., 2001). Specifically, JS0208MD was more effective than ASC alone in preventing increased expression of Il-1β although for other cytokines measured, there was no difference between ASC and JS0208MD. Future studies are needed to address the importance of ASC alone or whether equal or better efficacy can be acquired from combinations of compounds. These results are in contrast to recent findings where SVCT2 protein levels were significantly increased with LPS treatment (Portugal et al., 2017). These contradictions may in part be explained by different cell types used, dose range (100 ng to 1 μg/ml LPS) and investigation of mRNA expression vs. protein expression changes, though overexpression models in all of the cell types do seem to be protective against microglial reactivity. Other studies looking at SVCT2 protein expression in a stroke model (middle cerebral artery occlusion) also found upregulation in protein days 2–5 after injury in brain endothelial cells (Gess et al., 2011). However in this model, another group found decreased SVCT2 mRNA at 2 and 22 h at the infarct area, but increased expression in adjacent areas and in astrocytes specifically (Berger et al., 2003). The role of SVCT2 in inflammatory response is clearly complex and needs detailed characterization under different conditions and across time courses.

Taken together, these studies demonstrate that expression of the ASC transporter SVCT2 plays an important role in attenuating neuroinflammatory microglial responses, as evidenced by changes in morphology and inflammatory signaling both *in vivo* following mild traumatic brain injury and *in vitro* when cells were treated with LPS.

## 5. Conclusion

The present study tested the potential role of SVCT2 expression in microglia using *in vivo* and *in vitro* models of neuroinflammation. Both *in vivo* and primary *in vitro* data support a role for depleted SVCT2 expression and brain ASC in contributing to a microglial neuroinflammatory state. SVCT2^+/−^ microglial from tissue and cells showed the greatest range of response to injury or LPS challenge in comparison to wildtype controls. SVCT2-TG^BAC+^ microglia showed fewer changes overall compared to littermate controls. We also showed that a range of antioxidant compounds can attenuate cytotoxicity and proinflammatory signaling in a similar way to ASC. Overall, this work highlights the role ASC and its transporter SVCT2 may play in microglial neuroinflammatory response, providing further insight into the complex mechanisms driving microglial changes in neurological disorders.

## Supplementary Material

Marino Supplementary

## Data Availability

Data will be made available on request.

## Abbreviations:

ASC: Ascorbate
mTBI: mild Traumatic Brain Injury
SVCT2: Sodium-dependent vitamin C transporter 2
SVCT1: Sodium-dependent vitamin C transporter 1
LPS: lipopolysaccharide
ROS: reactive oxygen species
DHA: dehydroascorbic acid

## References

[R1] Aguzzi A, Barres BA and Bennett ML (2013) ‘Microglia: Scapegoat, Saboteur, or Something Else?’, Science, 339(6116), pp. 156–161. Available at: 10.1126/science.1227901. 23307732 PMC4431634

[R2] Arciniegas DB, , 2005. Mild traumatic brain injury: a neuropsychiatric approach to diagnosis, evaluation, and treatment. Neuropsychiatr. Dis. Treat 1 (4), 311–327. 18568112 PMC2424119

[R3] Batista CRA (2019) ‘Lipopolysaccharide-Induced Neuroinflammation as a Bridge to Understand Neurodegeneration’, International Journal of Molecular Sciences, 20(9), p. 2293. Available at: 10.3390/ijms20092293. 31075861 PMC6539529

[R4] Berger UV (2003) ‘Effect of middle cerebral artery occlusion on mRNA expression for the sodium-coupled vitamin C transporter SVCT2 in rat brain: Ascorbate transporter expression after ischemia’, Journal of Neurochemistry, 86(4), pp. 896–906. Available at: 10.1046/j.1471-4159.2003.01891.x. 12887688

[R5] Bolte AC and Lukens JR (2021) ‘Neuroimmune cleanup crews in brain injury’, Trends in Immunology, p. S1471490621000740. Available at: 10.1016/j.it.2021.04.003. PMC8165004 33941486

[R6] Brooks BL (2016) ‘Sex Differences and Self-Reported Attention Problems During Baseline Concussion Testing’, Applied Neuropsychology: Child, 5(2), pp. 119–126. Available at: 10.1080/21622965.2014.1003066. 25923339

[R7] Browne CA (2022) ‘Long-term increase in sensitivity to ketamine’s behavioral effects in mice exposed to mild blast induced traumatic brain injury’, Experimental Neurology, 350, p. 113963. Available at: 10.1016/j.expneurol.2021.113963. 34968423 PMC8858880

[R8] Cárcamo JM (2004) ‘Vitamin C Is a Kinase Inhibitor: Dehydroascorbic Acid Inhibits IκBα Kinase β’, Molecular and Cellular Biology, 24(15), pp. 6645–6652. Available at: 10.1128/MCB.24.15.6645-6652.2004. 15254232 PMC444845

[R9] Caye A, Axelrud LK and Salum GA (2018) ‘Traumatic brain injury and dementia’, The Lancet Psychiatry, 5(10), pp. 782–783. Available at: 10.1016/S2215-0366(18)30187-1. 30274671

[R10] Chatterjee S (1999) ‘Glutathione levels in primary glial cultures: Monochlorobimane provides evidence of cell type-specific distribution’, Glia, 27(2), pp. 152–161. Available at: 10.1002/(SICI)1098-1136(199908)27:2&lt;152::AID-GLIA5&gt;3.0.CO;2-Q. 10417814

[R11] Chiu C-C (2016) ‘Neuroinflammation in animal models of traumatic brain injury’, Journal of Neuroscience Methods, 272, pp. 38–49. Available at: 10.1016/j.jneumeth.2016.06.018. 27382003 PMC5201203

[R12] Consoli DC (2021) ‘Ascorbate deficiency decreases dopamine release in gulo ^–/–^ and APP/PSEN1 mice’, Journal of Neurochemistry, 157(3), pp. 656–665. Available at: 10.1111/jnc.15151. 32797675 PMC7882008

[R13] Dello Russo C (2018) ‘The human microglial HMC3 cell line: where do we stand? A systematic literature review’, Journal of Neuroinflammation, 15(1), p. 259. Available at: 10.1186/s12974-018-1288-0. 30200996 PMC6131758

[R14] Dinet V, Petry KG and Badaut J (2019) ‘Brain–Immune Interactions and Neuroinflammation After Traumatic Brain Injury’, Frontiers in Neuroscience, 13, p. 1178. Available at: 10.3389/fnins.2019.01178. 31780883 PMC6861304

[R15] Donat CK (2017) ‘Microglial Activation in Traumatic Brain Injury’, Frontiers in Aging Neuroscience, 9, p. 208. Available at: 10.3389/fnagi.2017.00208. 28701948 PMC5487478

[R16] Engelmann C, Weih F and Haenold Ronny (2014) ‘Role of nuclear factor kappa B in central nervous system regeneration’, Neural Regeneration Research, 9(7), p. 707. Available at: 10.4103/1673-5374.131572. 25206877 PMC4146279

[R17] Eyolfson E (2020) ‘Repetitive Mild Traumatic Brain Injuries in Mice during Adolescence Cause Sexually Dimorphic Behavioral Deficits and Neuroinflammatory Dynamics’, Journal of Neurotrauma, 37(24), pp. 2718–2732. Available at: 10.1089/neu.2020.7195. 32772786

[R18] Fendt M, Li L and Yeomans JS (2001) ‘Brain stem circuits mediating prepulse inhibition of the startle reflex’, Psychopharmacology, 156(2–3), pp. 216–224. Available at: 10.1007/s002130100794. 11549224

[R19] Feng J (2010) ‘Effect of therapeutic mild hypothermia on the genomics of the hippocampus after moderate traumatic brain injury in rats’, Neurosurgery, 67(3), pp. 730–742. Available at: 10.1227/01.NEU.0000378023.81727.6E. 20651628

[R20] Gess B (2011) ‘Sodium-Dependent Vitamin C Transporter 2 (SVCT2) Expression and Activity in Brain Capillary Endothelial Cells after Transient Ischemia in Mice’, PLoS ONE. Edited by Deli M, 6(2), p. e17139. Available at: 10.1371/journal.pone.0017139. 21347255 PMC3037964

[R21] Gessi S (2016) ‘The activation of μ-opioid receptor potentiates LPS-induced NF-kB promoting an inflammatory phenotype in microglia’, FEBS Letters, 590(17), pp. 2813–2826. Available at: 10.1002/1873-3468.12313. 27427408

[R22] Geyer MA (2001) ‘Pharmacological studies of prepulse inhibition models of sensorimotor gating deficits in schizophrenia: a decade in review’, Psychopharmacology, 156(2–3), pp. 117–154. Available at: 10.1007/s002130100811. 11549216

[R23] Gomez-Nieto R, Hormigo S and López DE (2020) ‘Prepulse Inhibition of the Auditory ´ Startle Reflex Assessment as a Hallmark of Brainstem Sensorimotor Gating Mechanisms’, Brain Sciences, 10(9), p. 639. Available at: 10.3390/brainsci10090639. 32947873 PMC7563436

[R24] Harrison FE and May JM (2009) ‘Vitamin C function in the brain: vital role of the ascorbate transporter SVCT2’, Free Radical Biology and Medicine, 46(6), pp. 719–730. Available at: 10.1016/j.freeradbiomed.2008.12.018. 19162177 PMC2649700

[R25] Harrison FE (2008) ‘Elevated oxidative stress and sensorimotor deficits but normal cognition in mice that cannot synthesize ascorbic acid’, Journal of Neurochemistry, 106(3), pp. 1198–1208. Available at: 10.1111/j.1471-4159.2008.05469.x. 18466336 PMC2575028

[R26] Harrison FE (2010) ‘Low vitamin C and increased oxidative stress and cell death in mice that lack the sodium-dependent vitamin C transporter SVCT2’, Free Radical Biology and Medicine, 49(5), pp. 821–829. Available at: 10.1016/j.freeradbiomed.2010.06.008. 20541602 PMC2916678

[R27] Harrison Fiona E. (2010) ‘Vitamin C distribution and retention in the mouse brain’, Brain Research, 1348, pp. 181–186. Available at: 10.1016/j.brainres.2010.05.090. 20570663 PMC2912448

[R28] Harrison FE (2012) ‘Increased Expression of SVCT2 in a New Mouse Model Raises Ascorbic Acid in Tissues and Protects against Paraquat-Induced Oxidative Damage in Lung’, PLoS ONE. Edited by Mukhopadhyay P, 7(4), p. e35623. Available at: 10.1371/journal.pone.0035623. 22558179 PMC3340390

[R29] Heldt SA, , 2014. ‘A novel closed-head model of mild traumatic brain injury caused by primary overpressure blast to the cranium produces sustained emotional deficits in mice’. Frontiers in Neurology 5. 10.3389/fneur.2014.00002. PMC3898331 24478749

[R30] Hirrlinger J (2000) ‘Microglial Cells in Culture Express a Prominent Glutathione System for the Defense against Reactive Oxygen Species’, Developmental Neuroscience, 22(5–6), pp. 384–392. Available at: 10.1159/000017464. 11111154

[R31] Ison JR, Allen PD, O’Neill WE, 2007. Age-related hearing loss in c57bl/6j mice has both frequency-specific and non-frequency-specific components that produce a hyperacusis-like exaggeration of the acoustic startle reflex. J. Assoc. Res. Otolaryngol. 8 (4), 539–550. 10.1007/s10162-007-0098-3. 17952509 PMC2538342

[R32] Kohl S, , 2013. Prepulse inhibition in psychiatric disorders – Apart from schizophrenia. J. Psychiatr. Res. 47 (4), 445–452. 10.1016/j.jpsychires.2012.11.018. Available at:23287742

[R33] Kumari V, , 2003. Sex differences in prepulse inhibition of the acoustic startle response. Pers. Individ. Differ. 35 (4), 733–742. 10.1016/S0191-8869(02)00266-0.

[R34] Kumari V, , 2008. A comparison of prepulse inhibition in pre- and postmenopausal women and age-matched men. Neuropsychopharmacology 33 (11), 2610–2618. 10.1038/sj.npp.1301670. 18216776

[R35] Langlois JA, Rutland-Brown W, Thomas KF, 2006. ‘Traumatic brain injury in the United States: emergency department visits, hospitalizations, and deaths’, Centers for Disease Control and Prevention, [Preprint]. National Center for Injury Prevention and Control, Atlanta.

[R36] Lehmann J, 1999. Sex differences in the acoustic startle response and prepulse inhibition in Wistar rats. Behav. Brain Res. 104 (1–2), 113–117. 10.1016/S0166-4328(99)00058-3. 11125729

[R37] Li Q, Barres BA, 2018. Microglia and macrophages in brain homeostasis and disease. Nat. Rev. Immunol. 18 (4), 225–242. 10.1038/nri.2017.125. 29151590

[R38] Lian H, Roy E, Zheng H, 2016. Protocol for primary microglial culture preparation. BIO-PROTOCOL 6 (21). 10.21769/BioProtoc.1989. PMC5669279 29104890

[R39] Livingston G, , 2020. Dementia prevention, intervention, and care: 2020 report of the lancet commission. Lancet 396 (10248), 413–446. 10.1016/S0140-6736(20)30367-6. 32738937 PMC7392084

[R40] Lu SC, 2013. Glutathione synthesis. Biochim. Biophys. Acta Gen. Subj. 1830 (5), 3143–3153. 10.1016/j.bbagen.2012.09.008. PMC3549305 22995213

[R41] Marino AL, Consoli DC and Harrison FE (2023) ‘Vitamin C and neuroinflammation’, in Vitamins and Minerals in Neurological Disorders. Elsevier, pp. 439–454. Available at: 10.1016/B978-0-323-89835-5.00028-4.

[R42] Martín-Sánchez F, , 2016. Inflammasome-dependent IL-1 β release depends upon membrane permeabilisation. Cell Death Differ. 23 (7), 1219–1231. 10.1038/cdd.2015.176. 26868913 PMC4946890

[R43] Maurya SK, , 2021. Microglia specific drug targeting using natural products for the regulation of redox imbalance in neurodegeneration. Front. Pharmacol. 12, 654489 10.3389/fphar.2021.654489. 33927630 PMC8076853

[R44] May JM, 2012. Vitamin C Transport and Its Role in the Central Nervous System. In: Stanger O (Ed.), Water Soluble Vitamins. Springer, Dordrecht, pp. 85–103. 10.1007/978-94-007-2199-9_6. Netherlands (Subcellular Biochemistry), Available at:PMC3725125 22116696

[R45] May JM, Qu Z, Mendiratta S, 1998. Protection and recycling of α-tocopherol in human erythrocytes by intracellular ascorbic acid. Arch. Biochem. Biophys. 349 (2), 281–289. 10.1006/abbi.1997.0473. 9448716

[R46] May JM, Qu Z, Li X, 2001. Requirement for GSH in recycling of ascorbic acid in endothelial cells 1 1Abbreviations: AFR, ascorbate free radical; BAECs, bovine aortic endothelial cells; CDNB, 1-chloro-2,4-dinitrobenzene; DHA, dehydroascorbic acid; HUVECs, human umbilical vein endothelial cells; KRH, Krebs-Ringer HEPES; and LDL, low density lipoprotein. Biochem. Pharmacol. 62 (7), 873–881. 10.1016/S0006-2952(01)00736-5. 11543722

[R47] Mielke MM, , 2022. Traumatic brain injury and risk of alzheimer’s disease and related dementias in the population. J. Alzheimer’s Disease: JAD 88 (3), 1049–1059. 10.3233/JAD-220159. 35723103 PMC9378485

[R48] Mikaelian DO, Warfield D, Norris O, 1974. Genetic Progressive hearing loss in the c57/m6 mouse: relation of behaviorial responses to cochlear anatomy. Acta Otolaryngol. 77 (1–6), 327–334. 10.3109/00016487409124632. 4835632

[R49] Morganti JM, , 2015. CCR2 antagonism alters brain macrophage polarization and ameliorates cognitive dysfunction induced by traumatic brain injury. J. Neurosci. 35 (2), 748–760. 10.1523/JNEUROSCI.2405-14.2015. 25589768 PMC4293420

[R50] Morrison H, , 2017. Quantitative microglia analyses reveal diverse morphologic responses in the rat cortex after diffuse brain injury. Sci. Rep. 7 (1), 13211. 10.1038/s41598-017-13581-z. 29038483 PMC5643511

[R51] Morrison HW, Filosa JA, 2013. A quantitative spatiotemporal analysis of microglia morphology during ischemic stroke and reperfusion. J. Neuroinflammation 10 (1), 782. 10.1186/1742-2094-10-4. PMC3570327 23311642

[R52] Mouzon BC, , 2018. Lifelong behavioral and neuropathological consequences of repetitive mild traumatic brain injury. Ann. Clin. Transl. Neurol. 5 (1), 64–80. 10.1002/acn3.510. 29376093 PMC5771321

[R53] Mullard A, 2018. Microglia-targeted candidates push the Alzheimer drug envelope. Nat. Rev. Drug Discov. 17 (5), 303–305. 10.1038/nrd.2018.65. 29700495

[R54] Mun GH, , 2006. Immunohistochemical study of the distribution of sodium-dependent vitamin C transporters in adult rat brain. J. Neurosci. Res. 83 (5), 919–928. 10.1002/jnr.20751. 16477646

[R55] Muzio L, Viotti A, Martino G, 2021. Microglia in neuroinflammation and neurodegeneration: from understanding to therapy. Front. Neurosci. 15, 742065 10.3389/fnins.2021.742065. 34630027 PMC8497816

[R56] Nayak D, Roth TL, McGavern DB, 2014. Microglia development and function. Annu. Rev. Immunol. 32 (1), 367–402. 10.1146/annurev-immunol-032713-120240. 24471431 PMC5001846

[R57] Pang KCH, , 2015. Long-lasting suppression of acoustic startle response after mild traumatic brain injury. J. Neurotrauma 32 (11), 801–810. 10.1089/neu.2014.3451. 25412226 PMC4449631

[R58] Parwani A, , 2000. Impaired prepulse inhibition of acoustic startle in schizophrenia. Biol. Psychiatry 47 (7), 662–669. 10.1016/S0006-3223(99)00148-1. 10745060

[R59] Pastor P, , 2013. ‘SVCT2 vitamin C transporter expression in progenitor cells of the postnatal neurogenic niche’, Frontiers in Cellular Neuroscience, 7. Available at: 10.3389/fncel.2013.00119. PMC3741466 23964197

[R60] Portugal CC (2017) ‘Caveolin-1-mediated internalization of the vitamin C transporter SVCT2 in microglia triggers an inflammatory phenotype’, Science Signaling, 10(472). Available at: 10.1126/scisignal.aal2005. 28351945

[R61] Portugal CC, Socodato R, Relvas JB, 2017b. The ascorbate transporter SVCT2 to target microglia-dependent inflammation. Oncotarget 8 (59), 99217–99218. 10.18632/oncotarget.22306. 29245893 PMC5725084

[R62] Portugal CC (2024) ‘Ascorbate and its transporter SVCT2: The dynamic duo’s integrated roles in CNS neurobiology and pathophysiology’, Free Radical Biology and Medicine, 212, pp. 448–462. Available at: 10.1016/j.freeradbiomed.2023.12.040. 38182073

[R63] Ritzel RM (2020) ‘Sustained neuronal and microglial alterations are associated with diverse neurobehavioral dysfunction long after experimental brain injury’, Neurobiology of Disease, 136, p. 104713. Available at: 10.1016/j.nbd.2019.104713. 31843705 PMC7155942

[R64] Rumsey SC (1997) ‘Glucose Transporter Isoforms GLUT1 and GLUT3 Transport Dehydroascorbic Acid’, Journal of Biological Chemistry, 272(30), pp. 18982–18989. Available at: 10.1074/jbc.272.30.18982. 9228080

[R65] Saban MR (2001) ‘Time course of LPS-induced gene expression in a mouse model of genitourinary inflammation’, Physiological Genomics, 5(3), pp. 147–160. Available at: 10.1152/physiolgenomics.2001.5.3.147. 11285368

[R66] Salazar K (2021) ‘SVCT2 Overexpression and Ascorbic Acid Uptake Increase Cortical Neuron Differentiation, Which Is Dependent on Vitamin C Recycling between Neurons and Astrocytes’, Antioxidants (Basel, Switzerland), 10(9), p. 1413. Available at: 10.3390/antiox10091413. 34573045 PMC8465431

[R67] Santa-Cecília FV (2016) ‘Doxycycline Suppresses Microglial Activation by Inhibiting the p38 MAPK and NF-kB Signaling Pathways’, Neurotoxicity Research, 29 (4), pp. 447–459. Available at: 10.1007/s12640-015-9592-2. 26745968

[R68] Savini I (2008) ‘SVCT1 and SVCT2: key proteins for vitamin C uptake’, Amino Acids, 34(3), pp. 347–355. Available at: 10.1007/s00726-007-0555-7. 17541511

[R69] Shao F (2022) ‘Microglia and Neuroinflammation: Crucial Pathological Mechanisms in Traumatic Brain Injury-Induced Neurodegeneration’, Frontiers in Aging Neuroscience, 14, p. 825086. Available at: 10.3389/fnagi.2022.825086. 35401152 PMC8990307

[R70] Simon DW (2017) ‘The far-reaching scope of neuroinflammation after traumatic brain injury’, Nature Reviews Neurology, 13(3), pp. 171–191. Available at: 10.1038/nrneurol.2017.13. 28186177 PMC5675525

[R71] Sinha SP (2017) ‘Startle suppression after mild traumatic brain injury is associated with an increase in pro-inflammatory cytokines, reactive gliosis and neuronal loss in the caudal pontine reticular nucleus’, Brain, Behavior, and Immunity, 61, pp. 353–364. Available at: 10.1016/j.bbi.2017.01.006. 28089558

[R72] Smith JA (2012) ‘Role of pro-inflammatory cytokines released from microglia in neurodegenerative diseases’, Brain Research Bulletin, 87(1), pp. 10–20. Available at: 10.1016/j.brainresbull.2011.10.004. 22024597 PMC9827422

[R73] Smith C (2013) ‘The neuroinflammatory response in humans after traumatic brain injury: Neuroinflammation after brain injury’, Neuropathology and Applied Neurobiology, 39(6), pp. 654–666. Available at: 10.1111/nan.12008. 23231074 PMC3833642

[R74] Son E-W (2004) ‘Vitamin C blocks TNF-α-induced NF-kB activation and ICAM-1 expression in human neuroblastoma cells’, Archives of Pharmacal Research, 27(10), pp. 1073–1079. Available at: 10.1007/BF02975434. 27518391

[R75] Subhramanyam CS (2019) ‘Microglia-mediated neuroinflammation in neurodegenerative diseases’, Seminars in Cell & Developmental Biology, 94, pp. 112–120. Available at: 10.1016/j.semcdb.2019.05.004. 31077796

[R76] Swerdlow NR (1999) ‘Sex differences in sensorimotor gating of the human startle reflex: all smoke?’, Psychopharmacology, 146(2), pp. 228–232. Available at: 10.1007/s002130051111. 10525760

[R77] Takahashi H (2011) ‘Prepulse Inhibition of Startle Response: Recent Advances in Human Studies of Psychiatric Disease’, Clinical Psychopharmacology and Neuroscience, 9(3), pp. 102–110. Available at: 10.9758/cpn.2011.9.3.102. 23429840 PMC3569113

[R78] Tsukaguchi H (1999) ‘A family of mammalian Na+-dependent L-ascorbic acid transporters’, Nature, 399(6731), pp. 70–75. Available at: 10.1038/19986. 10331392

[R79] Tweedie D (2020) ‘Time-dependent cytokine and chemokine changes in mouse cerebral cortex following a mild traumatic brain injury’, eLife, 9, p. e55827. Available at: 10.7554/eLife.55827. 32804078 PMC7473773

[R80] Verboon LN, Patel HC, Greenhalgh AD, 2021. ‘The Immune System’s Role in the Consequences of Mild Traumatic Brain Injury (Concussion)’. Frontiers in Immunology 12. 10.3389/fimmu.2021.620698. PMC7928307 33679762

[R81] Washington PM (2012) ‘The effect of injury severity on behavior: a phenotypic study of cognitive and emotional deficits after mild, moderate, and severe controlled cortical impact injury in mice’, Journal of Neurotrauma, 29(13), pp. 2283–2296. Available at: 10.1089/neu.2012.2456. 22642287 PMC3430487

[R82] Wiley JL (1996) ‘Reduced sensorimotor reactivity following traumatic brain injury in rats’, Brain Research, 716(1–2), pp. 47–52. Available at: 10.1016/0006-8993(96)00045-5. 8738219

[R83] Witcher KG (2021) ‘Traumatic Brain Injury Causes Chronic Cortical Inflammation and Neuronal Dysfunction Mediated by Microglia’, The Journal of Neuroscience, 41 (7), pp. 1597–1616. Available at: 10.1523/JNEUROSCI.2469-20.2020. 33452227 PMC7896020

[R84] Wofford KL, Loane DJ and Cullen DK (2019) ‘Acute drivers of neuroinflammation in traumatic brain injury’, Neural Regeneration Research, 14(9), pp. 1481–1489. Available at: 10.4103/1673-5374.255958. 31089036 PMC6557091

