# Supplementary material for: Modulation of microglia activation by the ascorbic acid transporter SVCT2
Source: Brain Behav Immun. Author manuscript; Available in PMC 2024 Oct 7. (PMC11458066; doi:10.1016/j.bbi.2024.07.003)
Supplement: Marino Supplementary [file NIHMS2022001-supplement-Marino_Supplementary.docx]

**Supplementary Materials**

**Supplemental** **Table 1:** List of primers used in the current study.

**Supplemental Figure 1**: Cortical ASC between sexes for each genotype mice.

A

**Supplemental Figure 1:** (A) ASC concentration in cortical tissue is not different according to sex. (n=9-10 per group, p’s>0.08)

**Supplemental Figure 2**: Representative Confocal Images of Primary Microglia

SVCT2^+/+^

Media

ASC

LPS

LPS+ASC


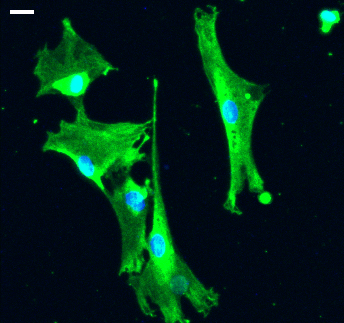

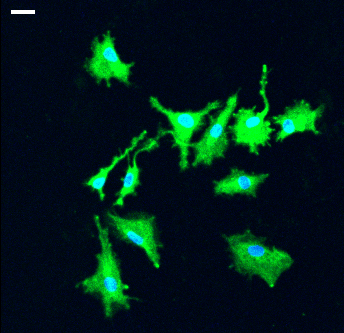

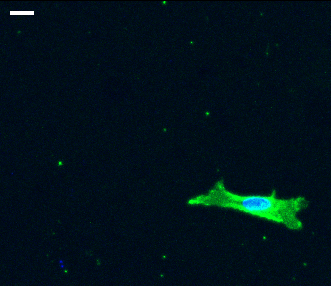

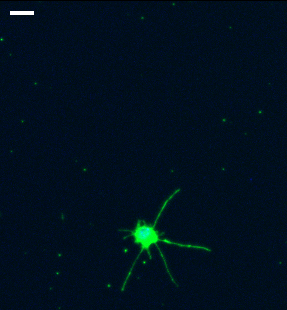

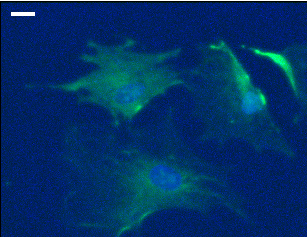

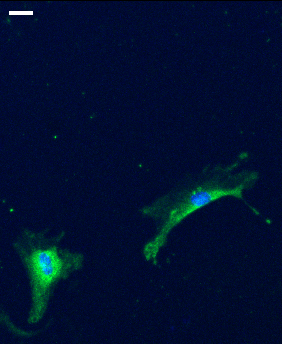

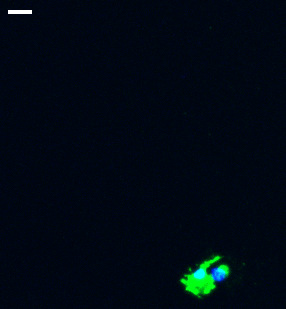

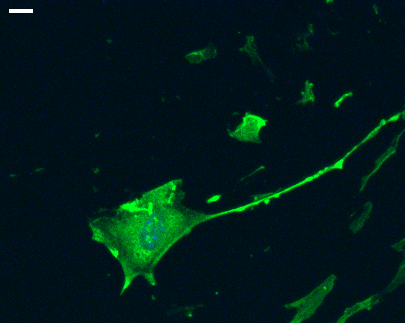

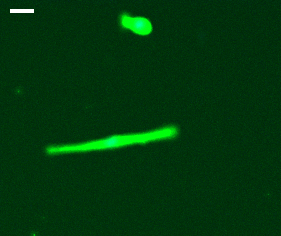

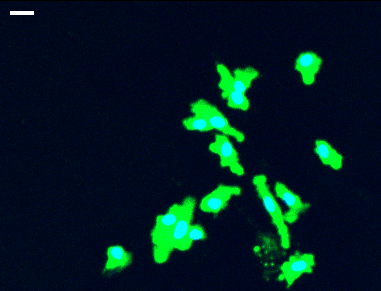

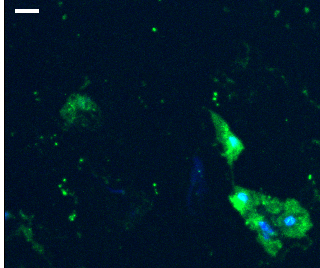

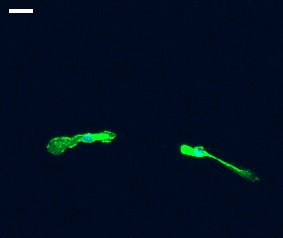

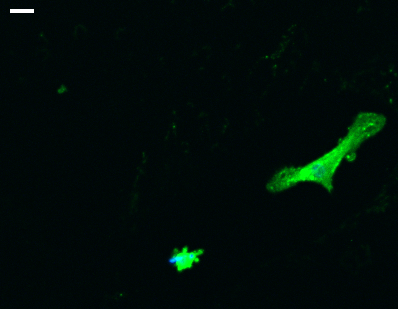

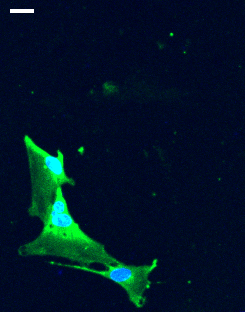

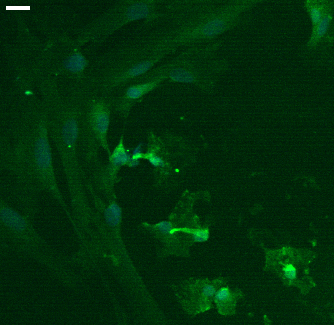

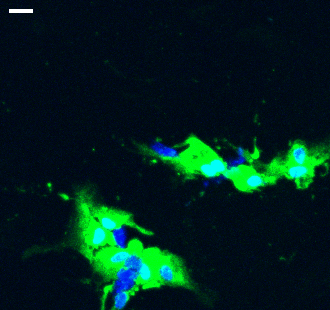


**Supplemental Figure 2:** Representative images of SVCT2^+/+^, SVCT2^+/-^, SVCT2-Tg^BAC-^, SVCT2-Tg^BAC+^ primary cultures stained with IBA-1 and DAPI. Scale bar represents 20µm.

**IBA-1**

**DAPI**

SVCT2^+/-^

SVCT2-Tg^BAC-^

SVCT2-Tg^BAC+^
